# When less is more: sketching with minimizers in genomics

**DOI:** 10.1186/s13059-024-03414-4

**Published:** 2024-10-14

**Authors:** Malick Ndiaye, Silvia Prieto-Baños, Lucy M. Fitzgerald, Ali Yazdizadeh Kharrazi, Sergey Oreshkov, Christophe Dessimoz, Fritz J. Sedlazeck, Natasha Glover, Sina Majidian

**Affiliations:** 1 Department of Fundamental Microbiology, UNIL, Lausanne, Switzerland; 2 Department of Computational Biology, UNIL, Lausanne, Switzerland; 3 SIB Swiss Institute of Bioinformatics, Lausanne, Switzerland; 4 Department of Endocrinology, Diabetology, Metabolism, CHUV, Lausanne, Switzerland; 5 Baylor College of Medicine, Houston, USA

## Abstract

The exponential increase in sequencing data calls for conceptual and computational advances to extract useful biological insights. One such advance, minimizers, allows for reducing the quantity of data handled while maintaining some of its key properties. We provide a basic introduction to minimizers, cover recent methodological developments, and review the diverse applications of minimizers to analyze genomic data, including de novo genome assembly, metagenomics, read alignment, read correction, and pangenomes. We also touch on alternative data sketching techniques including universal hitting sets, syncmers, or strobemers. Minimizers and their alternatives have rapidly become indispensable tools for handling vast amounts of data.

**Supplementary Information:**

The online version contains supplementary material available at 10.1186/s13059-024-03414-4.

## Introduction

Advances in computational and sequencing methods over the last two decades have propelled us into the genomics era [1], with databases like the European Nucleotide Archive increasing their assembled sequences and sequencing read collections by 100 and 100 million times, respectively, in that timespan [2]. Data in repositories now spans petabytes [3], decreasing costs and rising sequencing capabilities.

Genetic data is increasing in two dimensions. On one hand, moonshot initiatives with a “sequence everything” philosophy such as the Earth BioGenome Project [4] and the Tara Oceans Project [5] are sequencing a diverse range of species and microbial communities in a variety of environments [6]. On the other hand, initiatives such as the 1000 Genomes Project, the UK Biobank, and TOPMed aim to sequence hundreds of thousands of genomes from the same species [7, 8]. This trend has gained even more momentum with the advent of personalized medicine, where sequencing patients’ genomes is expected to become routine for diagnostics. For example, the “All of Us” Research Program aims to gather health and genetic data from one million people in the US [8, 9]. The abundance of genetic data presents a wide array of applications across many domains, including drug development or improving crop traits such as yield and resistance to climate change [3, 10, 11]. This wealth of sequencing data delivers many opportunities and challenges for using and storing all this information and developing scalable methods that can speed up its analysis.

One of the fundamental problems in bioinformatics is sequence comparison, which entails quantifying the similarity and dissimilarity between the sequences’ bases or amino acids and their order in sequences. It is key in processes such as identifying homologous genes or proteins [12, 13], genome assembly [14], and metagenomics species classification [15]. As the number of input sequences increases, the computational burden of pairwise comparisons increases quadratically, making handling the sheer amount of data more time-consuming and resource-intensive. To address this issue, various computational approaches have been developed to enable faster processing and comparison of large collections of sequencing data.

Traditionally, sequence comparison relied on alignment-based methods, which involve identifying the corresponding bases or amino acids in different sequences by maximizing a similarity score rewarding matches, and penalizing mismatches, insertions, or deletions [16]. For a wide range of scores, exact solutions can be computed using dynamic programming [17], but the time complexity is typically quadratic in the length of the sequences or worse. This is too slow for many contemporary applications, which involve analyzing sequences of billions of base pairs. Faster approximation algorithms have been devised, such as the well-known BLAST [18], Diamond [19], or MMseqs [20] tools. They use various heuristics as shortcuts, including some of the techniques discussed below, but they are still considered “alignment-based” in that they retain some dynamic programming approach at their core.

To avoid computationally costly alignments, many alignment-free methods have been developed which improve memory requirements and time complexity when handling large amounts of data [21]. Two important concepts applied in many alignment-free sequence comparison methods are *k-*mers (also known as n-grams, the “K-mer definition and properties” section) and graph-based representations (e.g., de Bruijn graphs, the “Representing de Bruijn graphs” section). Sketching methods are also a popular alternative. In the broader sense, sketching is a technique to create a reduced representation of the data that retains important properties and can be used to replace the original data in some applications [6, 22]. Some sketching examples are locality-sensitive hashing, minimizers, or bloom indexes [6, 22, 23].

**Fig. 1:**
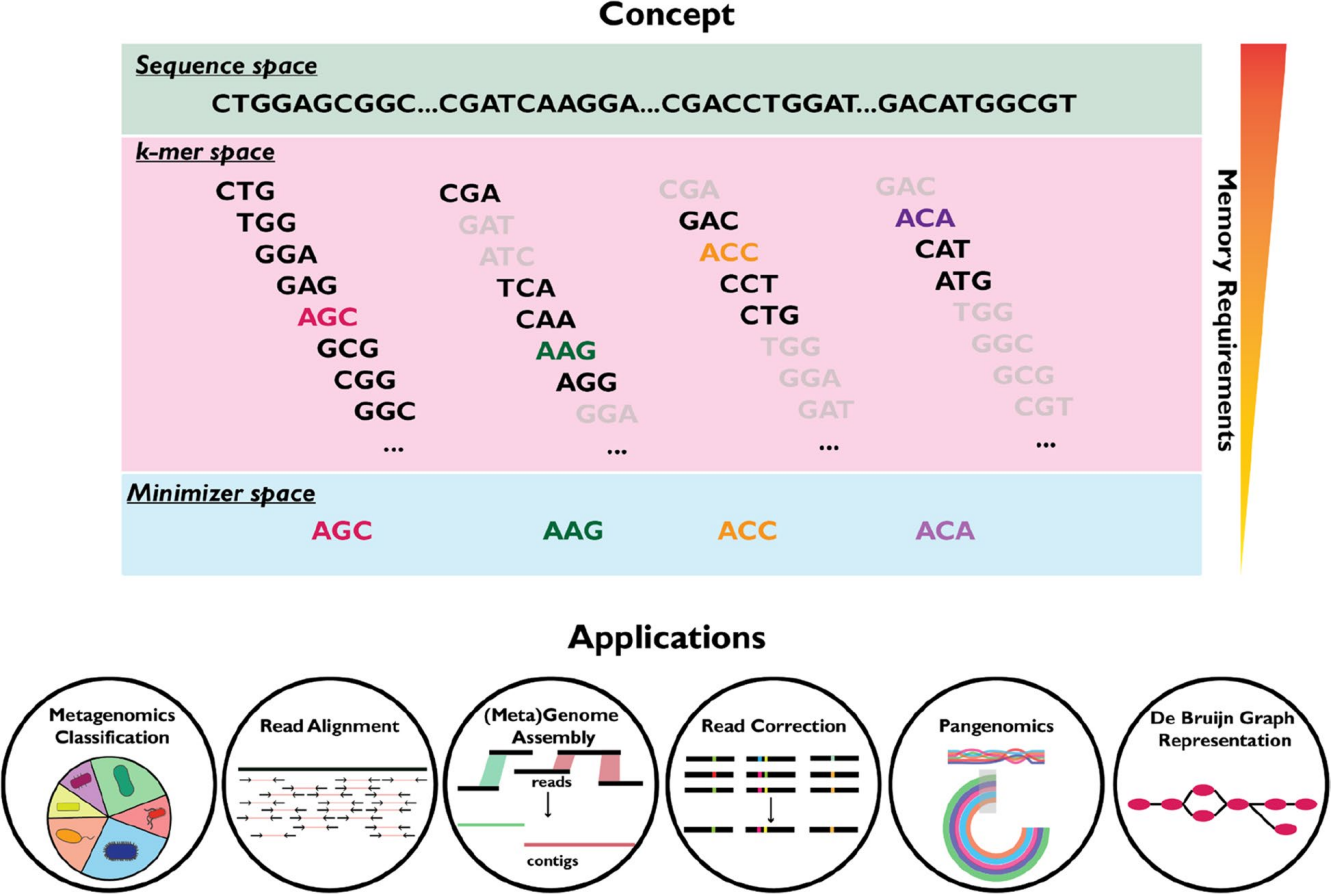
The concept and applications of minimizers. *K*-mers are fixed-length substrings of a sequence and are used to analyze genomic sequences. The minimizer approach reduces the computational requirements by selecting only a representative *k-*mer from a group of adjacent *k*-mers. Minimizers are useful in a diverse range of applications in bioinformatics and computational biology including read alignment, read correction, de Bruijn graph representation, genome assembly, pangenomics, metagenomics classification and assembly, and beyond

In this review, we focus on the highly efficient sketching approach of minimizers. In recent years, minimizers have emerged as a powerful approach to handle the ever-increasing amount of sequencing data efficiently, while maintaining or even surpassing the accuracy of traditional methods. Nevertheless, the function and advantages or disadvantages of minimizers compared to more traditional approaches is often unclear. To provide a comprehensive understanding of minimizers, we begin with explaining *k-*mers, which serve as the foundation for minimizers, and explore the minimizers scheme definition and properties (the “Background” section). Then, we discuss six of the most notable applications of minimizers in genomics (Fig. 1): read alignment (the “Read alignment” section), read correction (the “Read correction” section), representing de Bruijn graphs (the “Representing de Bruijn graphs” section), genome assembly (the “De novo genome assembly” section), pangenomes (the “Pangenomes” section), and metagenomics (the “Metagenomics” section) as well as briefly touch on minimizer alternatives (the “Minimizer alternatives” section). Finally, we conclude and discuss the future of minimizers in genomics.

## Background

### *K*-mer definition and properties

*K-*mers are useful for analyzing large DNA or RNA sequences, as they allow storing and manipulating these sequences using smaller, more manageable substrings [3, 22]. This reduces the time and memory complexity of analyzing large amounts of sequences by decreasing the search space, paving the way for more efficient algorithms for tasks such as genome assembly, mapping gene expression data, and sequence classification. Based on the assumption that similar subsequences that can be aligned with alignment-based methods also share *k-*mers, we can identify similar subsequences by comparing the *k-*mers in sequences. Moreover, we can store repeated *k-*mers only once, possibly accompanied with their positions or their frequencies, resulting in a more compact storage. Storing sequences using *k*-mers comes with a loss of information because it only informs us about each *k-*mer rather than the whole sequence, but they may retain sufficient information for many purposes as described later in this review [3]. Although storing many *k*-mers can be computationally challenging [3, 24], utilizing *k-*mer-based methods is generally more efficient than alignment-based methods [16].

Some essential *k-*mers definitions and notions are needed before we introduce minimizers. A *k*-mer is a substring or a “word” of length *k* present in a longer sequence *S*. Two contiguous *k*-mers in a string share *k*-1 characters. It follows that if |*S*| represents the length of the sequence, the maximum number of* k*-mers in *S* is |*S*|− *k* + 1, which can be approximated to |*S*|, assuming that *k* is much smaller than |*S*|. Naively, storing all* k*-mers of *S* would require a space of O(|*S*|*k), which is more than the sequences themselves [25].

Nevertheless, storing* k*-mers can be more space-efficient than storing complete sequences, because the maximum number of different possible *k*-mers is |Σ|^k^, Σ being the alphabet [26]. For example, if *k* is 2 and the alphabet is the DNA bases Σ = {A, C, G, T}, there will be at most 4^2^ possible *2*-mers: B_2_ = {AA, AC, AG, AT, CA, CC, CG, CT, GA, GC, GG, GT, TA, TC, TG, TT}. Storing these* 2*-mers occupies less space than storing the complete string if the string *S* is longer than 16 bases. Note that the number of possible unique *k*-mers increases exponentially with *k*, quickly exceeding |*S*|. However, in practice, the number of observed *k*-mers is bounded by |*S*|− *k* + 1 and many *k*-mers are typically repeated [3]. As a result, typical *k* values are kept within the range of 20 to 200 and each distinct observed *k*-mer is stored only once, along with their frequencies and/or their positions, depending on the application.

Exploiting *k-*mers was a breakthrough in handling sequence data. However, as data grows, the linear increase in storage demand becomes impractical, necessitating more efficient ways to handle genomics data [25]. This is especially true as many genomic comparisons do not require such level of detail as the *k*-mer approach provides. One such way that has emerged is minimizers, which is based on *k-*merization of the data. Minimizers achieve faster processing and reduced memory usage by working with only a subsample of the *k-*mers.

### Minimizers

The minimizers scheme is a sequence analysis approach to create approximate representations of sequences, or sketches, which occupy a reduced space in comparison to the sequences themselves. Minimizers were originally introduced by Roberts et al. [25] to reduce the number of stored *k*-mers needed to assemble genomes and to reduce computations for sequence comparison compared to traditional methods like BLAST [18]. Interestingly, the concept of minimizers referred to as “winnowing” had already been independently developed for fingerprinting documents and detecting plagiarism [27, 28].

A minimizer is a selected representative *k-*mer from a group of adjacent *k*-mers. However, this approach is useful only if two substrings with an exact match end up sharing at least one of the representative *k-*mers. For instance, choosing every *k-*th *k-*mer as a minimizer is inadequate because two strings with a long exact match would only share a *k-*mer if it starts at the same position or at a position multiple of *k*. Therefore, to ensure functionality, minimizer schemes must be defined by complying with specific properties to guide the selection of representative *k-*mers, discussed below [22, 25].

### Parameters and properties of a minimizers scheme

**Fig. 2:**
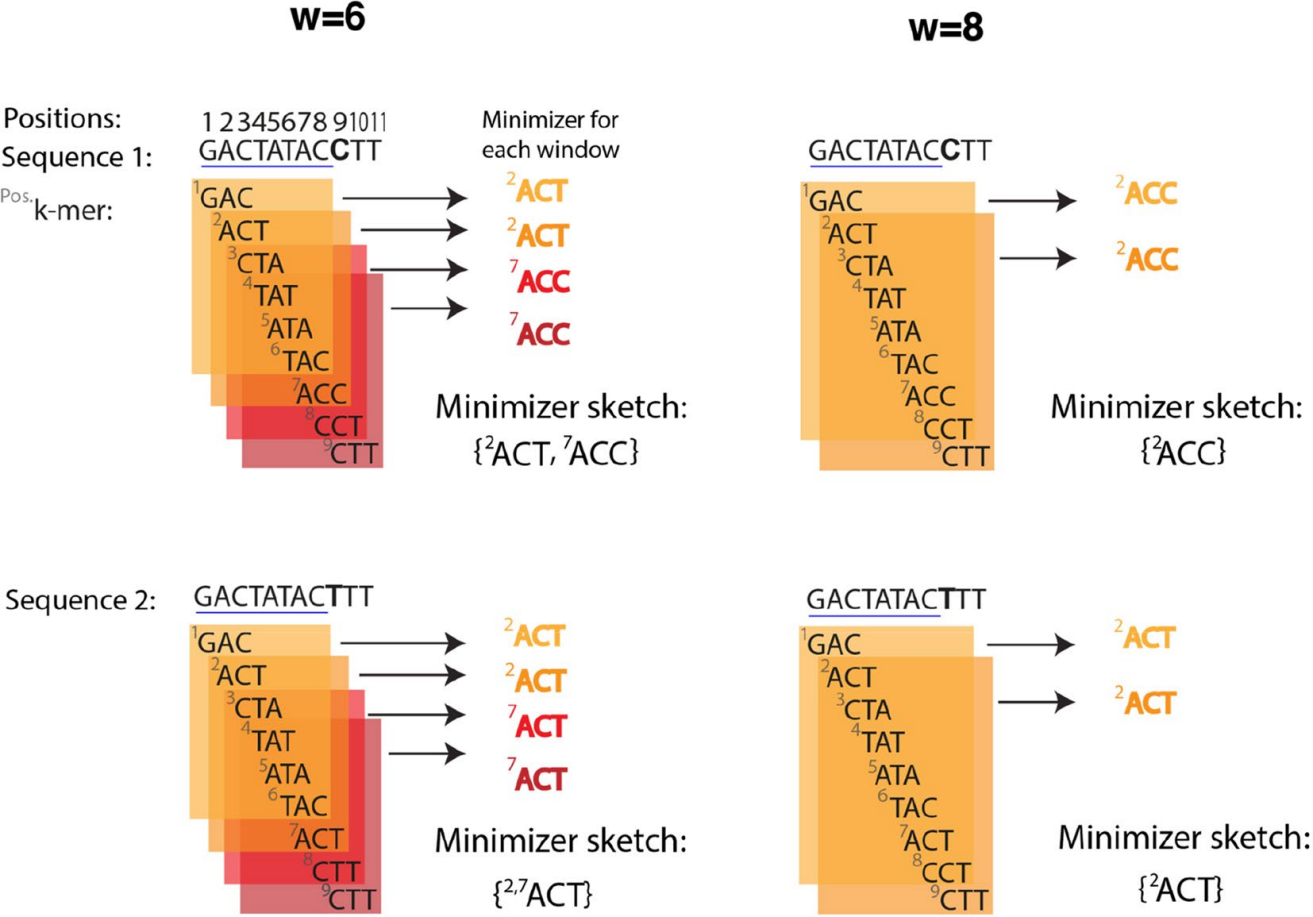
Implementation of two minimizer schemes with differing *w* values (left and right) for two sequences with an exact match of length 8 shown in blue underline. The parameter *k* = 3 and the ordering (lexicographic) are constant. The length of each sequence is |*S*|= 11 having 9 ( =|*S*|− *k* + 1) *k*-mers. Each box represents the window with size *w* (6 or 8), corresponding to the starting positions of the window’s *k*-mers which covers *w* + *k* − 1 bases (8 or 10, respectively). For sequence 1, using *w* = 6, the selected minimizer in the first window (partly covering the underlined exact match) is ACT starting at position 2. The same minimizer, ACT, is also selected for sequence 2 using *w* = 6. Since the exact match length is 8 (≥ *w* + *k* − 1), the first property of minimizers schemes is fulfilled, and the same minimizer is chosen for both sequences, representing the exact match. However, when using *w* = 8 (right), the match length is < *w* + *k* − 1. Thus, there is no guarantee of sharing a minimizer and a different *k-*mer is chosen for each sequence in this example. Note that for the second window in sequence 2, we break the tie between ACTs starting at position 2 and 7 with the leftmost position; this happens for both* w* = 6 and*w* = 8. The density of the minimizers scheme for sequence 1 using *w* = 6 is 2/9, as two minimizers are chosen in total: ACT (position 2, for the two first windows) and ACC (position 7, for the two last windows), and the density for sequence 2 is also 2/9 using *w* = 6. With *w* = 8, the density for both sequences is 1/9

A minimizers scheme is defined by three parameters: the *k*-mer length (*k*), the window size (*w*), and the ordering. A window with size of *w* corresponds to *w* consecutive* k*-mers covering a substring of length *w* + *k* − *1* from which a *k*-mer is selected as the representative called the minimizer. Minimizers are chosen based on an ordering (i.e., sorting) of the *k-*mers, such as lexicographic [25, 28]. By choosing the “smallest” *k*-mer as the minimizer (Fig. 2), the selection is not based on the *k*-mer’s position but rather based on the sequence content. Choosing minimizers begins with the first substring starting at position *S*[1], selecting a minimizer among the *w* consecutive *k-*mers starting at positions *S*[1], *S*[2], …, *S*[*w*] (considering 1-based indexing). Then, it proceeds sequentially, identifying a minimizer for the second window in the range [2, *w* + 1], then the third window [3, *w* + 2], and so on all the way to [|*S*|− *w* − *k* + 2, |*S*|− *k* + 1]. The set of all minimizers obtained in this way are the minimizers of sequence *S*. Because neighboring substrings have overlapping windows, their associated minimizers are often identical; as a result, the set of minimizers of *S* tends to be much smaller than the set of *k*-mers of *S*. Of note, the choice of *k* and *w* varies among applications. For example, *k* = 15 and *w* = 10 are used for long, noisy read alignment in *minimap2* discussed in the “Read alignment” section.

A minimizer scheme has two important consequent properties. The first property is that “two sequences with an exact match of minimum length *w* + *k* − 1 will share a minimizer” [25]. In other words, any matches of length ≥ *w* + *k* − 1 will be represented in the selected minimizer while shorter matches might not be, depending on their ordering position (Fig. 2). The second property is that “the maximum distance between two consecutive selected *k*-mers is *w*,” as at least one *k*-mer must be selected per window. In case of ties—where two *k-*mers in a window have the same order—solutions include storing all tied *k*-mers [22] or selecting based on additional criteria, such as choosing the *k-*mer at the leftmost position [25].

### The importance of ordering

The *k*-mer ordering parameter is crucial in constructing the minimizers, as it significantly influences their performance. Performance can be measured by density, defined as the ratio of selected *k-*mers among all *k-*mers of a given substring, where lower density indicates higher efficiency [22, 27]. Since different orderings lead to different selected minimizers and thus varying densities, the *k-*mer ordering approach has a large impact on the performance of the minimizer scheme and should be tailored for each application.

Using the lexicographic order for strings with frequent “A”s can lead to selecting multiple consecutive *k-*mer minimizers (consider e.g., AAATCGT with *k* = 3, *w* = 5), thereby leading to an undesirable increase in density. Roberts et al. [25] recommend an ordering strategy that favors choosing rare *k*-mers as minimizers, resulting in lower density. For DNA sequences, this can be achieved by prioritizing less frequent bases or by selecting *k*-mers with a higher count of these bases [25].

**Fig. 3:**
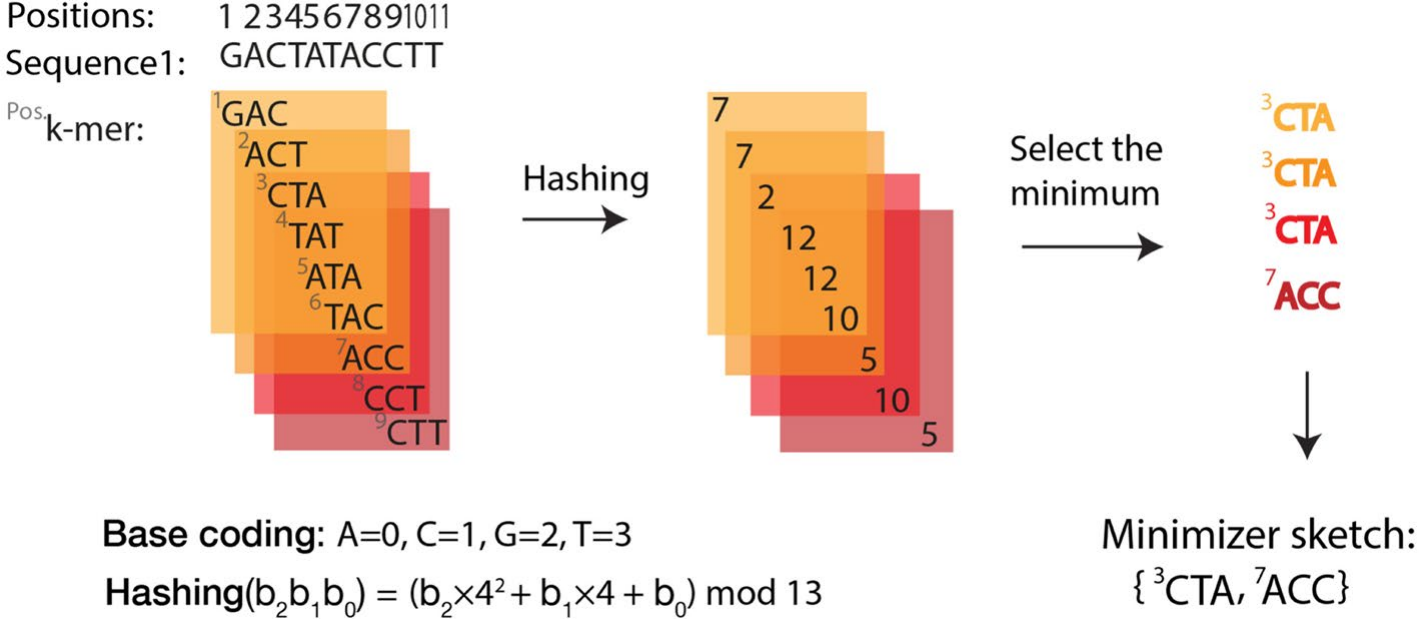
An example implementation of a minimizers scheme using a hash function for ordering. In this case, the hash function calculates the remainder of the values assigned to each *k-*mer divided by 13. The *k*-mer with the lowest hash value in a window is selected as the minimizer. For the last window, we break the tie between ^7^ACC and ^9^CTT with hash value of 5, by selecting the one starting at the leftmost position resulting in ^7^ACC

An alternative to lexicographic ordering is using functions to assign numerical values to *k*-mers [29]. A hash function transforms a given string of arbitrary size (*k*-mers in our case) into a fixed-sized value. In doing so, the resulting representation will typically occupy less space [29] (e.g., hashing into 32-bit values [30] results in space saving for genomic *k*-mers for *k* > 16 assuming a 2-bit encoding per nucleotide). See Fig. 3 as an example of using a hash function for defining a minimizer scheme.

Several studies have focused on optimizing ordering and devising new data structures and schemes to achieve minimizers with higher efficiencies and lower densities (fewer selected *k-*mers) [22]. Theoretically, density ranges from 1/*w* (since at least one *k-*mer is chosen for every *w* letters based on the property 2) to 1 (where all *k-*mers are selected). The optimal minimum of 1/*w* is only achieved when *k* is large  [22, 31], and the interest lies in constructing a minimizer with a density within a constant factor, i.e., O(1/*w*) for any k*.* With lexicographic ordering, minimizers can achieve such density, but with large *k* values (≥ log_|Σ|_(w)-c for a constant c), which might not be desirable [32]. However, random ordering can result in a lower density than that of the lexicographic ordering. Thus, random ordering (implemented with pseudo-random hash functions) is usually used in practice [28, 31–33].

The expected density, defined as the expectation of density over all possible sequences when bases are chosen independently with equal probability, is used to evaluate minimizer ordering. Using a random ordering and a window size of *w* ≪|Σ|^*k*^, the expected density is proven to be *2/(w* + *1)*, with some other assumptions which might not hold in practice [27]. Zheng et al. provided explicit conditions only on *k* (i.e.,* k* ≥ (3 + ε)log_|Σ|_(*w* + 1)) for the expected density of 2/(*w* + 1) + o(1/*w*) [32]. The added term o(1/*w*) relates to the probability of having two identical *k*-mers in a random window of *w* which equals to |Σ|^−*k*^ or equivalently 1/*w*^3+ε^ = o(1/*w*^*3*^) under the mentioned assumption on *k*. See [31, 34, 35] for asymptotic analysis.

Recent investigations indicate that ordering algorithms can achieve a density value of 1.8/(*w* + 1) [36], well below the originally proposed lower bound of 2/(*w* + 1) [22, 25]. Of note, several studies have developed new data structures to improve the density, some of which are described in the ”Minimizer alternatives” section. In addition to density, other metrics to analyze sketching schemes including conservation [23], repetitiveness [37] coverage and sketch score [38] have also been suggested. For a more in-depth review of the algorithmic aspects of minimizers, see [39]. In short, it is increasingly clear that minimizers are a powerful tool to improve memory efficiency and runtime in several applications, and research on their optimal design is still ongoing.

## Minimizer applications

Given the promising advances of minimizers compared to *k*-mer approaches, we next review their wide-ranging applications. Table 1 summarizes various applications and programs that utilize minimizers to increase their speed and memory efficiency, highlighting the broad applications of minimizers across different research fields. Key applications include read alignment, read correction, genome assembly, pangenomes, and metagenomics.

**Table 1:** Bioinformatics tools that use minimizers categorized in seven fields, namely, read alignment, read correction, de Bruijn graph (dBG) representation, genome assembly, pangenomes, metagenomics classification, and assembly

Main application	Name	Description	Citation
Read alignment	*minimap2*	Uses a seed-chain-align procedure by collecting minimizers	[40, 41]
	*GraphAligner*	Long-read aligner to genome graphs using minimizers	[42]
	*LRA*	Aligns long reads to a reference genome	[43]
	*Chromap*	Aligns chromatin profiles using minimizers	[44]
	*Winnowmap and Winnowmap2*	Weighted-minimizer sampling algorithm that builds on top of *minimap2*	[45, 46]
Read correction	*Miniscrub*	Convolutional neural network-based method for removing low-quality nanopore read segments	[47]
	*VeChat*	Correcting errors in long reads using variation graphs	[48]
	*isONcorrect*	Long-read error correction	[49]
	*Minirmd*	Removing duplicate and near-duplicate reads	[50]
de Bruijn graph (dBG) representation	*BCALM2*	Parallel dBG compaction	[51]
	*Bifrost*	Parallel dBG compaction	[52]
	*GGCAT*	Parallel *k*-mer enumeration and dBG compaction	[53]
	*Fulgor*	ccdBG representation for alignment-free sequence matching	[54]
De novo genome assembly	*rust-mdBG*	De novo genome assembly from minimizer-space dBG	[55]
	*MBG*	De novo genome assembly from minimizer-based dBG	[55, 56]
	*LJA*	Long-reads de novo genome assembly	[57]
	*ntJoin*	Reference-based genome assembly	[58]
	*Wengan*	Hybrid short- and long-reads de novo genome assembly	[59]
Pangenomes	*Minigraph*	Pangenome construction from multiple genomes (Eukaryote-vertebrate focus) and sequence to graph aligner	[60]
	*Giraffe*	Fast mapping of short-reads to pangenome (Eukaryote-vertebrate focus)	[61]
	*PGR-TK*	Pangenome construction and analysis using sparse hierarchical minimizers (Human focus)	[62]
	*Pandora*	Pangenome construction and analysis. Capture core and accessory genes as well as variants (Bacteria focus)	[63]
Metagenomics classification	*Kraken and Kraken2*	Metagenomics classifier by minimizer with improved memory requirements	[64, 65]
	*K2Mem*	Classifier based on *kraken2* with improved memory and classification time	[66]
	*MetaMaps*	Analyzer for long-read metagenomics data	[67]
Metagenomics assembly	*MetaProb2*	Genome binning method using minimizers to assemble reads	[68]

### Read alignment

Read alignment involves placing and comparing DNA or RNA sequencing reads to a reference genome or transcriptome. The goal is to identify the best match between a given read and the reference, since this is the best hypothesis for where the read originated. A naive approach to read alignment would be to use brute force by checking all possible positions, but this is impractical due to the vast number of reads generated by sequencing technologies. Additionally, read alignment can be computationally demanding because the reference genome may contain repeated sequences where a read can map to with equal probability. Furthermore, sequencing errors and true genetic variability within a sample can introduce differences between the read and its matching location within the reference.

Read alignment is a crucial step in various genomic pipelines including identifying and studying genetic variations. A wrongly placed or misaligned read often leads to a falsely-identified variant with consequences for downstream analyses [69, 70]. To address speed and accuracy of read alignments, over 100 methods have been developed [69]. The list includes the Burrows-Wheeler Aligner (BWA) [71] and Bowtie [72] for short DNA reads, BLASR [73] and BWA-SW [74] for long DNA reads, and STAR [75] for RNA-seq reads. For comprehensive reviews of tools for read alignment, see [69, 76]. Here, we focus only on methods for read alignment that employ minimizers.

Many alignment tools benefit from a seed-and-extend or seed-chain-extend approach designed for short or long reads: The goal of seeding is to find small exact matches between the read and the reference which are then chained together using dynamic programming [18, 71, 77–79].

**Fig. 4:**
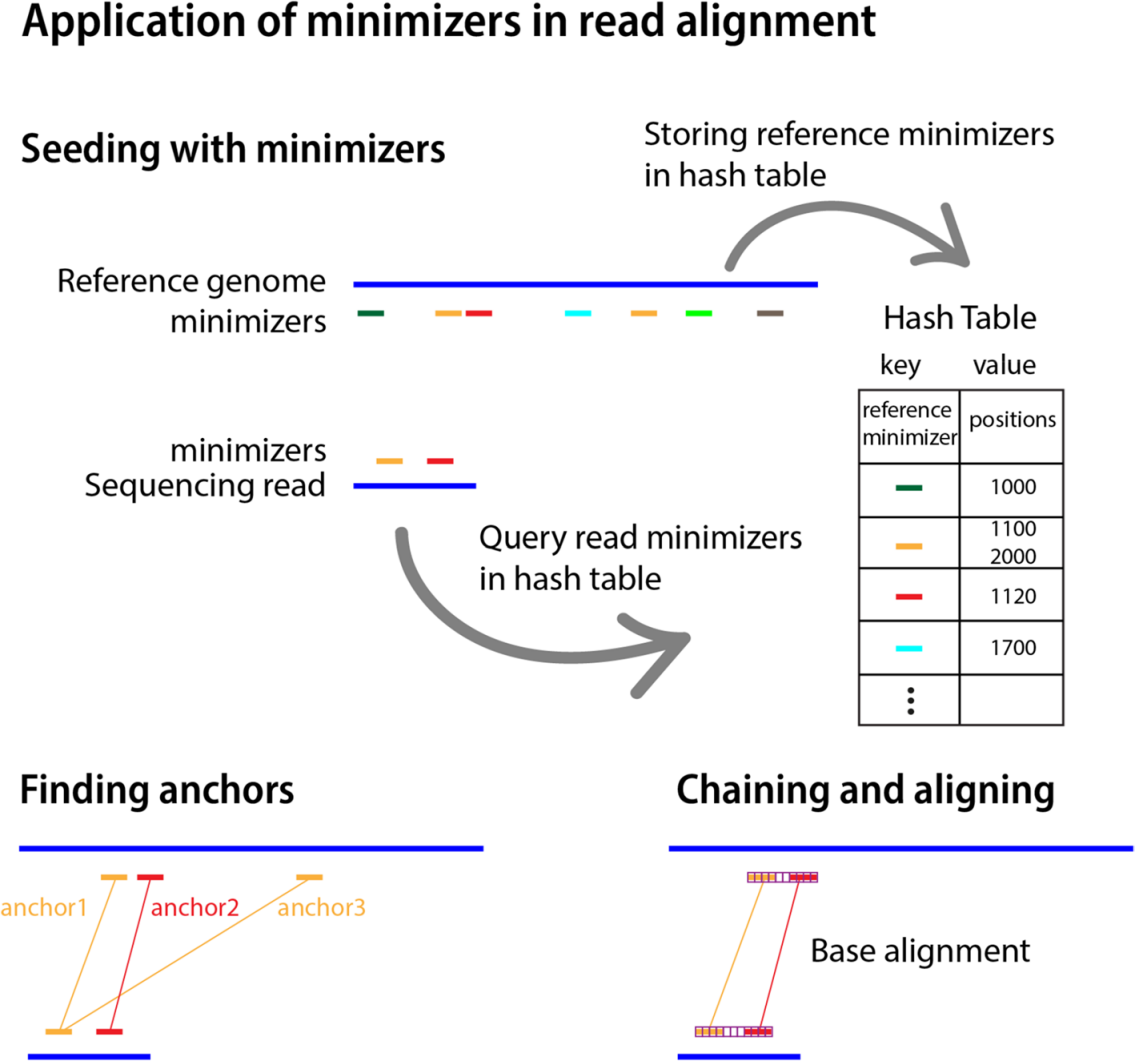
Application of minimizers in read alignment. A typical read aligner that follows the seed-chain-align approach first finds reference minimizers and stores them in a hash table. Seeds are substrings (minimizers) from the reference or the read. Seeds that match between the read and the reference are called anchors, which are found by querying the read minimizers in the hash table. Then, anchors are chained together and finally bases are aligned

To find exact matches, different approaches including Burrows-Wheeler transform (BWT), suffix arrays, or minimizers are used (reviewed in [80]). *Minimap* [81] is an alignment tool for nucleotide sequences whose improved version, *minimap2*, was released in 2018 [40] and quickly became one of the leading tools for read alignment. This tool benefits from a seed-chain-align approach, employing minimizers to identify initial exact matches, seeds. *Minimap2* first finds minimizers within the reference, with computation time being linear in terms of the length of the reference [81]. These are stored in a hash table where the minimizer’s hash values (obtained with a hash function) are the keys and the minimizer locations are the values (Fig. 4). For query sequences (reads), seeds are also formed from their minimizers. Anchors are found by exact matching read seeds to positions in the reference hash table. An anchor in *minimap2* denotes a pair of starting positions, indicating a range on the reference sequence that matches a range on the query sequence. Then, a chain is formed from a set of collinear anchors using a dynamic programming approach to maximize matching bases between anchors considering a customized cost function of gap length [40, 41]. Finally, to create alignments, *minimap2* applies dynamic programming to extend chains and to fill regions between neighboring anchors inside the chain. Minimizers here help to avoid an exhaustive per-base search of seed matches.

Furthermore, *minimap2* uses various heuristics for optimization. To avoid wrong anchors in a chain, which could appear due to local homology and sequencing errors, *minimap2* filters out anchors that lead to insertions and deletions (> 10 bp) or a long gap at the end of the chain. While this alleviates issues with misplaced anchors, it is unable to fix all such errors. Nonetheless, in comparison with alternative aligners, *minimap2* shows superior accuracy and speed, sometimes at the cost of memory [40]. In 2021, Li improved *minimap2* by using more minimizers (previously it kept only low-occurrence minimizers) and refining its chaining algorithm by changing the alignment scoring function. This improvement addressed challenges like un- or mis-aligned reads in highly repetitive regions and the alignment of sequences with long insertions/deletions (indels) [41].

Building on top of *minimap2*, *Winnowmap* introduced a weighted-minimizer sampling algorithm [45]. *Minimap2* ignores frequent minimizers because minimizers from repetitive regions are sampled more often, which artificially increases seed hits. However, this results in overturning the property 1 of minimizers and in accuracy reduction. To tackle this challenge, *Winnowmap* performs minimizer sampling by considering a weight for each *k-*mer; the higher the weight of the *k-*mer, the more likely it is to be selected. Repetitive *k*-mers with frequency above 1024 are given a weight of 1/8, while other *k*-mers are given a weight of 1. With this approach, property 1 remains true for this weighted-minimizer scheme while avoiding excessive false matches. This method leverages *minimap2*’s techniques for anchor chaining and gapped alignment for read alignment, achieving up to 50% lower memory usage while maintaining a similar runtime to *minimap2*. *Winnowmap2* (Table 1) uses the same seeding approach of *Winnowmap* and improves on *minimap2*’s extending using heuristic to address allelic biases. *Winnowmap2* can efficiently map long reads to repetitive reference sequences and has improved accuracy in variant calling of the Genome in a Bottle samples [82] than other long-read mappers such as *Winnowmap*, *minimap2* and *NGMLR* [46].

*LRA* is a method for aligning long sequencing reads to a reference genome, which it accomplishes in four main steps: seed sequence matching, clustering, chaining, and refinement [43]. It tries to find the solution to seed chaining with a concave gap function to differently penalize opening or extending a gap. After finding anchors using minimizers, *LRA* filters out unreliable ones by partitioning them into clusters using a greedy approach. These represent approximate intervals on the query and target that are aligned. These clusters form fragments represented as diagonal lines in a 2D cartesian space correlating the sequences of the read and the reference genome. The chain with the lowest score is found in a more efficient manner than the traditional O(*n*^2^), achieving O(*n* log^2^*n*) time complexity, where *n* is the number of fragments. This chaining leads to refined alignments that resulted in higher sensitivity of variant discovery compared to *minimap2* and *NGMLR* with comparable runtime [43].

*MashMap* [83] formulates the read mapping problem using the Jaccard similarity coefficient of *k-*mers between the read and its mapping region on the reference. The Jaccard is defined as the ratio of the intersection size over the union size of two sets. It is estimated using *MinHash* with the smallest set of hash values of *k*-mers of two sequences [57]. Due to the expensive computational costs for comparing a read and the reference sequence, *MashMap* uses *MinHash* on minimizers rather than all* k*-mers. Recently, *MashMap2* [84] has also been released for whole-genome alignments using the same minimizer-centric approach.

To study chromatin organization and accessibility, analyzing ChIP-seq or ATAC-seq data is now becoming routine. In chromatin profiling, the standard approach is to start with short-read aligners like *BWA-MEM* or *Bowtie2*, followed by sorting reads and removing duplicates. This is an inefficient process since base-level alignment is not needed for most chromatin analysis. Moreover, several isolated tools are used involving high reading and writing operations on files. *Chromap* is a fast, integrated tool for analyzing chromatin profiles, adopting minimizer indexing from *minimap2* to find seeds, but with a different approach for seeding and alignment [44]. *Chromap* follows a similar approach to *minimap2* for chaining anchors and generates alignment candidates. Finally, a bit-parallel algorithm is used to find the best alignment candidate with the lowest edit distance. *BWA-MEM*, *minimap2*, and *Chromap* all performed similarly, with 98% accuracy for simulated 100 and 150-bp paired-end data. On smaller 50-bp paired-end data, *BWA-MEM* and *Chromap* had a similar accuracy of 96%, while *minimap2* lags slightly with performance ranging between 94 and 96% [44].

Read alignment can also be performed on genome graphs. *GraphAligne*r is a minimizer-based method for long read alignment to graphs [42]. The input to *GraphAligner* could be any bidirected graphs (modeling double helix DNA which could be traversed in two directions) including de Bruijn (see the “Representing de Bruijn graphs” section), variation, and pangenome graphs (see the “Pangenomes” section). *GraphAligner* uses a seed-and-extend method where seeds are identified by exact matching each read to the sequences of nodes in the graph. Of note, only seed hits that are entirely contained in a node are considered. To find the matches, a minimizer index is built from the graph by sliding a window through the node’s sequence and finding the smallest *k-*mers using the BBHash function. When aligning reads to a linear reference, seeds are chained by solving the co-linear chaining problem via computation of the distance between seeds. However, in a, graph the distance between seeds is ambiguous due to the presence of branching paths. This is addressed by chaining superbubbles, which are defined as acyclic subgraphs having one entrance and exit node and some internal nodes. Superbubbles are chained when one end node is the start of another. This can be used to assign linear position to seed hits which is then treated as linear sequence alignment. Then, each sequence is extended using a banded dynamic programming approach and Viterbi’s algorithm to decide the end of a read alignment. When traversing node sequences, overlap between nodes (for example for a de Bruijn graph where nodes overlap *k-1* bases) should be considered in the matching process. Performance wise, *GraphAligner* is more than ten times faster than the VG tool [42, 85]. For the case of aligning simulated data on linear reference, *minimap2* and *GraphAligne*r have similar accuracy (95%); however, *GraphAligner* has three times the runtime of *minimap2*. Despite being slower than *minimap2*, it is still faster than linear mappers such as *BWA* [42].

Overall, read aligner methodologies have been improved by using minimizers, particularly the chaining algorithm developed in *minimap2* which revolutionized the field. *Minimap2* has been built upon and compared to many tools and methodologies as evident in this section and the “Pangenome” section.

### Read correction

Long reads from sequencing technologies like Pacific Biosciences (PacBio) and Oxford Nanopore Technologies (ONT) are powerful tools for genome and metagenome assembly as well as transcriptomics. Their length can reach several kilobases, allowing a single read to span low-complexity regions which would be otherwise difficult to assemble or to incorporate an entire RNA transcript end-to-end [86–88]. However, long reads have higher error rates compared to their short counterparts [89–91]. This can hinder the assembly process, increase computational loads, and introduce biases in downstream analyses [89, 90, 92]. To tackle this problem, de novo error correction can be performed using multiple sequence alignment (MSA) among reads to infer the correct sequence using a consensus approach. However, the quadratic nature of comparing reads all-vs-all can be computationally prohibitive [93]. Thus, some of the recent tools for de novo error correction (Table 1) use minimizers to reduce the computational requirements of MSA.

*Miniscrub* and *VeChat* are two tools that use minimizers-based *minimap2* to find overlapping reads using shared minimizers and perform de novo error correction of long error-prone reads [47, 48]. *Miniscrub* uses *minimap2* to efficiently perform all-vs-all read alignments. Then, it generates a “pileup” image that visualizes the aligned reads, where each column of pixel denotes a position on the reference, and each row corresponds to an individual read aligned to that position. The pileup image pixels contain relevant information in Red–Green–Blue format, such as matched minimizers, distance between minimizers, and base quality scores. This image serves as input for a convolutional neural network pre-trained by the developers on sets of reads from known reference genomes to learn the correlation between a read’s level of support from other reads, as represented in the pileup image, and its accuracy. Leveraging this learning, the neural network predicts high-quality read fragments based on the supporting reads for the fragment’s minimizers. Finally, a user-defined threshold is used to filter out low-quality segments, yielding shorter high-quality reads.

*VeChat* uses *minimap2* to perform all-vs-all read alignments in metagenomics samples. Then, it divides the alignments into 500-bp fragments and uses a variation graph (VG; see the “Pangenomes” section) approach to correct the reads. This graph is used to prune low support edges that likely correspond to sequencing error, while maintaining edges that represent true haplotype variation within the samples.

Minimizers are also useful for error correction in long-read transcriptomics data. The *isONcorrect* method uses minimizers to splice ONT reads into non-overlapping fragments that begin and end at different minimizers of the read [49]. Fragments sharing prefix and suffix minimizers are likely to share homologous sequences. Thus, these fragments are clustered together and aligned independently to perform error correction following a consensus approach. By using minimizers, this approach reduces the computational load of comparing a large number of reads. The authors show that *isONcorrect* reduces the mismatch rate of long reads from the *Drosophila* genome from a median of 7% to a median of 1.1% [49].

Minimizers can also be used to remove duplicate and near-duplicate reads. In turn, this can reduce computational resources in downstream applications by decreasing the amount of redundant information in the dataset. *Minirmd* is a read deduplication tool that performs de novo clustering of reads in function of the shared minimizers [50]. Briefly, reads sharing a minimizer in the same minimizer position are clustered in the same group. *Minirmd* performs multiple rounds of clustering using minimizers of varying *k* values (*k*-minimizers) to prevent the clustering of near-duplicates in separate groups caused by mismatches between specific *k*-minimizers reads. Then, reads within the same cluster are compared pairwise to identify duplicates, near-duplicates, and reverse complements. Finally, the read with the best quality is retained. *Minirmd* was able to remove on average 3% more near-duplicates than other deduplication tools like CD-HIT-DUP, Fulcrum, and MarDRe, while being faster and using less memory [50].

### Representing de Bruijn graphs

In this section, we will review the application of minimizers in optimizing the representation of de Bruijn graphs (dBGs). A (node-centric) dBG is a directed graph where the edges are represented by all distinct *k*-mers extracted from an input sequence (e.g., sequencing reads or genomes [94]). Nodes within this graph correspond to the *k-1* suffixes and prefixes of the *k*-mers. Edges connect nodes found in a *k*-mer [95, 96]. dBGs are fundamental data structures in computational genomics, used in applications such as genome and metagenome assembly (the “De novo genome assembly” and “Metagenomics” sections) and pangenome representation (the “Pangenomes” section) as well as sequence identification or matching [54].

The construction of dBGs from a sequence can be summarized into four main steps: (1) *k*-mer enumeration, (2) graph construction, (3) graph compaction, and (4) graph cleaning. Firstly, the set of distinct *k*-mers is extracted from the input sequence and the graph is constructed as previously explained. Then, all paths with all but the first and last nodes having an in- and out-degree of 1 (known as unitigs) are compacted into a single node to obtain a compacted dBG (cdBG). Finally, all paths with low support (i.e., representing rare *k*-mers in the read dataset) are pruned because they probably originate from sequencing errors. Moreover, bubbles in the graph, often caused by polymorphisms or repeated regions, may be collapsed based on criteria like read coverage and path length, depending on the specific dBG implementations [96]. Of note, when a dBGs is constructed starting from a collection of datasets, nodes can be labeled with additional information, such as the sample of origin for a given *k*-mer, resulting in what is known as a “colored” dBG [97, 98].

The primary advantage of dBGs lies in the compact representation of the input sequences, as repeated substrings are represented only once in the graph, significantly reducing the space required for storage. However, storing the dBG of large sequences can have a substantial memory footprint, especially in its initial and uncompacted form [99]. This represents the main bottleneck to the scalability of dBG construction. For instance, the dBG representation of the 20-Gbp white spruce genome required around 4.3 TB of memory [100]. Complicating matters, dBG construction is not easily parallelizable [101]. Given the exponential increase of sequencing data handled by researchers, there has been a concerted effort to leverage minimizers to improve the efficiency of dBG construction, with particular emphasis on *k*-mer enumeration and graph compaction.

One way to optimize dBG construction is to parallelize the process. *BCALM2* [51] executes parallel graph compaction by categorizing *k*-mers into disk buckets utilizing *s*-mer minimizers selected in the *k-1* prefixes and suffixes of the *k*-mers. *K*-mers with distinct minimizers at the two ends are assigned to different disk-buckets. Subsequently, *k*-mers within the same disk-bucket undergo compaction by grouping minimizers to identify overlaps. The relatively small size of the buckets allows for parallel compaction through an in-memory algorithm. The original *k*-mers are then utilized to merge unitigs from different disk-buckets, effectively reuniting *k*-mers that were initially assigned to two separate disk-buckets. This merging operation is also conducted in parallel, since the algorithm ensures that strings requiring reunification (i.e., sharing the same *k*-mer at one extremity) are grouped into the same partition. This allows each partition to be processed independently. This final step enables the reconstruction of maximal unitigs, resulting in a cdBG. Compared to previous endeavors [102, 103], *BCALM2* was shown to reduce the time and the memory required for *k*-mer counting and graph compaction of the White Spruce and Loblolly Pine genomes by 1 to 2 orders of magnitude [51].

*Bifrost* utilizes minimizers for the construction and indexing of colored and compacted dBGs (ccdBGs) in a highly parallelized manner. The algorithm integrates minimizers within the framework of Blocked Bloom Filters (BBFs), a data structure designed for memory-efficient membership queries of an element in a set [52]. During the insertion of a *k*-mer into BBFs, *Bifrost* leverages the hash value of its minimizer to determine the appropriate BBF block for the *k*-mer. This ensures that sequences preceding or succeeding a particular *k*-mer are included in the same BBF block. Since *k*-mers in different BBF blocks can be handled independently, this optimization significantly contributes to the parallelization of unitig extraction, dBG compaction and navigation, decreasing the runtime and memory required. *Bifrost* was shown to construct a ccdBG of around 110,000 *Salmonella* strains in 93 h using about 100 GB of memory [52].

*GGCAT* represents a significant improvement over *Bifrost*, achieving a 5 × faster construction of a ccdBG from a collection of 100 datasets of human genome sequences and a 480 × faster query for *k* = 27 [53]. The key innovation of *GGCAT* lies in integrating *k*-mer enumeration with unitig construction in a highly parallelizable manner. This begins by partitioning the input sequence into substrings having *k-2* characters overlap and having all (*k*-1)-mers within each substring share the same minimizer. These substrings are further extended with one linking base in each direction, to ensure that consecutive substrings overlap by exactly *k* bases. Subsequently, the substrings are grouped based on their minimizers. For each group of substrings, *k*-mer counting is performed concurrently. This grouping guarantees that overlapping *k-*mers are stored and processed independently, allowing parallelization of the unitig construction process. The algorithm then initiates unitig construction by starting with each *k*-mer and extending it both left and right. This extension process involves identifying potential overlaps and matches with neighboring *k*-mers in the same group. Reaching the linking bases during extension signifies the endpoint of the unitig in that direction. The corresponding *k*-mer and the unitig will be stored as a tuple. Finally, these tuples are processed based on overlapping *k*-mers (found in another group) to generate maximal unitigs by iteratively merging unitigs [53].

More recently, *SSHash* has been developed for constructing dBGs with high scalability [104, 105], building on ideas introduced by *BLigh*t [106]. *BLight* is an efficient exact data structure that allows membership queries of a *k-*mer and its associated information. It splits unitigs of the cdBG into super-*k*-mers, which are sequences composed of consecutive *k-*mers sharing the same minimizer. The set of *k-*mers of a super-*k*-mer is indexed using a minimal perfect hash function associating identifiers to *k-*mers used during querying. Such a function bijectively maps each of *i* inputs (keys) into a unique integer in the range of {0..i-1} without collisions.

*SSHash* improves storage efficiency by storing absolute offsets that point to the positions in the genomic sequences where each super-*k-*mer starts, instead of indexing the concatenation of super-*k*-mers. This improves space efficiency since, in practice, several super-*k*-mers are small. Additionally, *SSHash* leverages the fact that minimizers have a skewed distribution: most minimizers appear only once, while a few appear multiple times. Frequent minimizers are managed with a minimal-perfect Hash function. This ensures efficient and constant-time lookups (i.e., reporting the unique identifier of *k*-mer in the set) when the number of offsets represented by a minimizer is large. Infrequent minimizers are managed by a regular lookup procedure where entries are directly accessed and iterated through until a match is found. This dual strategy enhances both storage efficiency, retrieval, and manipulation of genomic data, which is crucial for tasks like sequence alignment. Of note, *SSHash* has been extended to represent weights (i.e., abundance counts) [105] and attracted interests in the literature for indexing genomes [31, 107], and *k*-mer/unitig membership queries [108].

*Fulgor* integrates *GGCAT* with the *SSHash* data structure to optimize the representation of ccdBGs which could be used for alignment-free matching of metagenomic sequences against a reference database (aka pseudo-alignment) [54]. Initially, *GGCAT* constructs a ccdBG from a given set of reference sequences. *Fulgor* employs the *SSHash* data structure to efficiently store the ccdBG unitigs. By leveraging *SSHash*, *Fulgor* efficiently stores ccdBG components in a compact way, optimizing memory usage and enabling fast queries for consecutive *k-*mers, which often share the same minimizer. To be more exact, unitigs are sorted by their color IDs and are stored via *SSHash*. This enables *Fulgor* to compute the color of each unitig using a rank query on the bit vector where 1 shows a change in color of consecutive unitigs (rank-one query returns the sum of the 1 s in a vector). *Fulgor* was shown to build a ccdBG from 150,000 *Salmonella* strains in under 5 h, utilizing approximately 137 GB of RAM, resulting in an index with size of only 70 GB, which was further reduced to 7.5GB  [98] (much smaller than those of the competitors). This represents a significant improvement over the above-mentioned performance reported for *Bifrost* on a similar dataset.

In summary, the optimization of dBG construction through the use of minimizers has led to significant advancements in genomic data analysis. Innovations such as *BCALM2*, *Bifrost*, and *GGCAT* have demonstrated how minimizers can enhance parallelization and efficiency in *k*-mer enumeration and graph compaction. Additionally, the development of data structures like SSHash has optimized the storage and query of dBGs. The integration of these advancements in tools like *Fulgor* exemplifies the potential for further improvements in dBG representation, particularly in the efficient handling and querying of large-scale datasets.

### De novo genome assembly

In this section, we review the application of minimizers to the problem of de novo genome assembly to achieve contiguous, high-quality assemblies of large genomes in a computationally efficient fashion. De novo genome assembly deals with the problem of reconstructing a consensus sequence* G* of length |G| from a randomly sampled set of reads of length *r*, where *r* < <|G| [109, 110]. This problem has also been extended to assemble all haplotypes of a diploid or polyploid species [111–113].

A straightforward approach to de novo genome assembly entails searching for overlaps between reads to infer a consensus set of sequences that approximates the original genome. This approach is known as overlap layout consensus (OLC) [114, 115], which is particularly good at handling sequencing errors or genomic regions with high heterozygosity by allowing overlaps with mismatches [101]. However, finding overlaps between reads usually needs all-vs-all comparison resulting in quadratic time in the number of reads. Thus, the more reads that are present in a dataset, the more computationally prohibitive it becomes to find overlaps and a consensus [116]. This is notably the case when handling millions of short reads, a scenario which is typical of the output generated by Illumina sequencing technologies.

OLC remains a popular approach for genome assembly, particularly when dealing with long-read technologies such as ONT and PacBio with higher error rates. In these cases, heuristics are often employed to mitigate the all-vs-all alignment bottleneck. For example, *Hifiasm* leverages minimizers to find overlaps among reads and perform read correction, making it capable of generating highly contiguous and haplotype-resolved assemblies of large eukaryotic genomes using PacBio HiFi reads [117].

On the other hand, dBGs have emerged as a powerful alternative to OLC for the assembly problem. Indeed, following dBG construction (the “Representing de Bruijn graphs” section), contigs (i.e., contiguous segments of the genome being assembled) are extracted from the simplified paths of the compacted and cleaned dBG [96]. The number of nodes of an error-cleaned dBG saturates at higher sequencing depths but mainly depends on the size of the sequenced genome [116]. Thus, dBGs lead to a progressive reduction in computational time and memory required to assemble a genome [96, 100], bringing the time complexity of genome assembly down to O(|G|) where |G| is the genome size. This makes dBGs more suitable to assemble deeply sequenced genomes, especially when working with short-reads technologies.

Since their introduction, dBG-based assemblers performed well with bacterial and small eukaryotic genomes but needed a substantial amount of time and memory when handling large eukaryotic genomes [101]. For example, *ABySS*, one of the pioneering dBG-based genome assemblers capable of assembling mammalian-sized genomes [118], needed 87 h on a cluster of 21 eight-core machines, each one equipped with 16 GB of RAM, to assemble a human genome in > 4 million contigs. Moreover, dBGs rely on perfect *k-1* overlaps between *k*-mers. This poses a challenge when dealing with long error-prone reads, as it gives rise to branches in the assembly graphs [119]. Thus, while the construction of dBGs is theoretically dependent on the genome’s size, the inevitable inclusion of sequencing errors can inflate the graph size.

The challenges in assembling large and complex genomes pushed researchers to find ways to optimize dBG construction by optimizing *k*-mer enumeration and reducing the size of the dBG to be stored in memory [116, 120]. This approach was pioneered by the developers of the assembly software *SparseAssembler* [121], which builds sparse dBGs by storing only a subset of *k*-mers evenly distributed across the input reads. This approach (conceptually similar to minimizers) allows to preserve the overall graph structure while storing only a small fraction of the nucleotides from the input data, thereby decreasing memory usage and speeding up graph construction. Indeed, *SparseAssembler* is reported to assemble a 370-Mbp rice plant genome in 5 h, reaching a memory peak of 5 GB. They showed a notable reduction compared to other popular assemblers at the time, such as *AbySS*, which required 13 h and 69 GB of RAM to complete the same task with comparable assembly quality [121].

Following *SparseAssembler*, new tools optimized the construction of sparse dBGs by opting for a minimizer-centric approach. This approach is particularly powerful when combined with long error-prone sequencing reads of large genomes, where the number of *k*-mers to be stored in memory becomes a serious burden on the performance of the assembler.

**Fig. 5:**
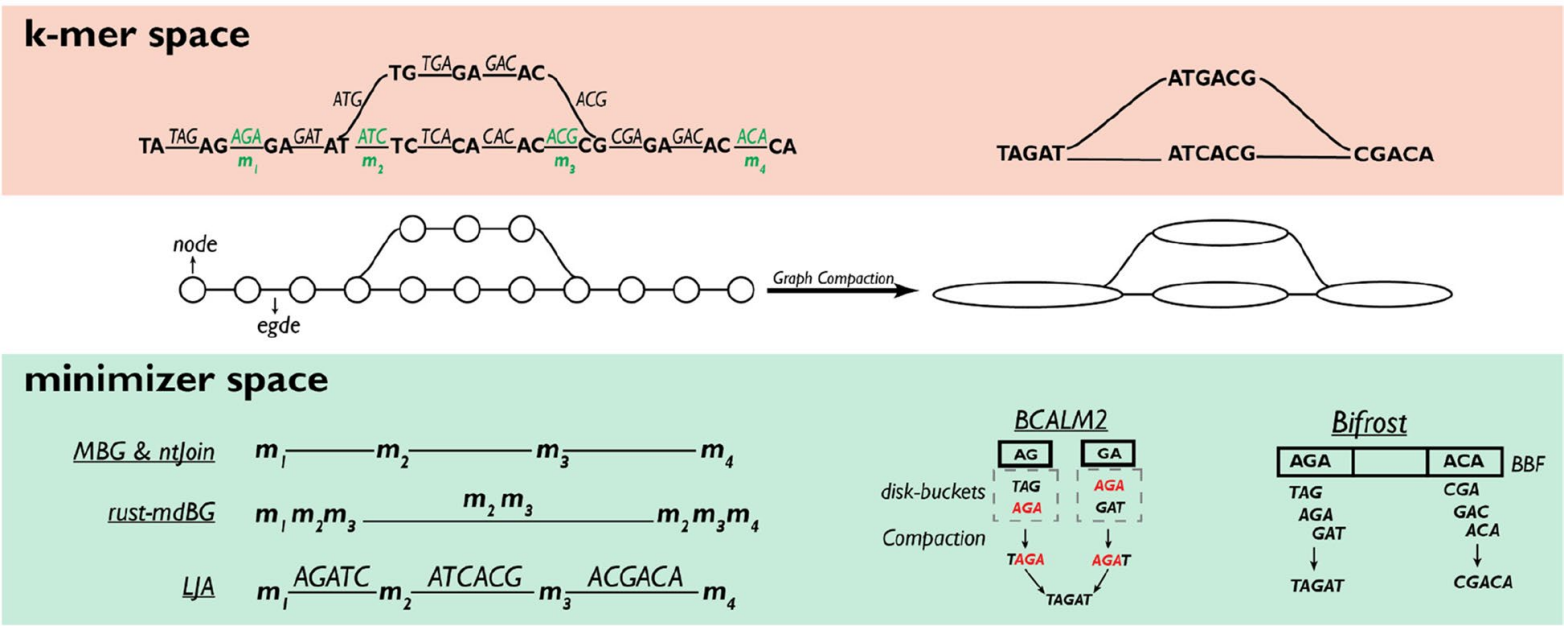
Implementation of minimizers in the construction and compaction of de Bruijn graphs (dBGs). Traditionally, a dBG is a directed graph where the edges are represented by all distinct *k*-mers extracted from the input reads. Nodes within this graph correspond to the *k-1* suffixes and prefixes of the *k*-mers which are connected by edges if they are in a *k*-mer. To optimize dBG construction, *MBG* and *ntJoin* employ minimizers as nodes, connecting adjacent minimizers with edges. Similarly, *LJA* incorporates “splits” as edges representing substrings between pairs of consecutive minimizers in the input reads. *rust-mdBG* utilizes tuples of *k′* minimizers as nodes (*k′* = 3 in this example), connecting nodes with overlaps of *k′-1*. Following graph construction, compaction is crucial for reducing dBG size for efficient memory storage. *BCALM2* and *Bifrost* leverage minimizers to parallelize graph compaction. *BCALM2* categorizes k-mers into disk-buckets based on suffix and prefix minimizers, while *Bifrost* adds k-mers to a blocked bloom filter according to the hash value of their minimizer. These data structures enable the parallel inference of maximal unitigs, enhancing the overall efficiency of the compaction process

The *MBG* tool identifies minimizers in the input sequences and stores their position on the reads. A dBG is constructed by using the minimizers as nodes and connecting them with edges if they are adjacent in a read [55, 56] (Fig. 5). Following graph cleaning and compaction, the dBG is converted back to the original base pair sequence. Similarly, *rust-mdBG* identifies minimizers from the original reads and subsequently scans the reads to pinpoint their positions. Then, it creates a set of tuples, each containing a specified number (*k′*) of adjacent minimizers. The order of these minimizers in a tuple is determined by their relative positions on the reads. These minimizers can appear multiple times within the same tuple or across different tuples, mirroring their occurrences in the original reads. In the resulting dBG, these tuples become the nodes, with edges established between nodes if there is a *k′*-1 overlap between the corresponding tuples [75]. The minimizer-space dBG construction results in a notable reduction in graph size, given that the graph selectively retains only the bases linked with minimizers. This focused representation provides an effective strategy for capturing crucial genomic information while minimizing data storage. Finally, it enables streamlined graph compaction and cleaning processes before inferring the final contigs. This is achieved by concatenating the read sequences spanned by the minimizers within the minimizer-space dBG. For example, r*ust-mdBG* assembled the human genome in 10 min using 10 GB of RAM with 8 threads using high-fidelity (HiFi) long reads.

At the time of its introduction, the La Jolla Assembler (*LJA*) was shown to achieve the more contiguous assembly of the human genome using HiFi reads, generating fivefold fewer misassemblies (i.e., incorrectly assembled sequences) than other software such as *hifiasm* and *hiCanu* [57]. *LJA*’s approach involves extracting minimizers from input reads, including the *k*-mer suffixes and prefixes of each read in the minimizer set to ensure that overlapping reads share a minimizer. Subsequently, a dBG is constructed in the minimizer space by defining “splits,” which are substrings between pairs of consecutive minimizers in the reads. These splits serve as edges connecting minimizers (nodes). Following dBG construction, LJA generates sequences known as “disjointing” via a random walk through the graph. Although disjointing may not directly correspond to sequences in the original genome, they efficiently preserve all the *k*-mers from the original read set in a reduced number of sequences. This feature allows them to be manipulated to construct a more time- and memory-efficient cdBG from *k*-mers extracted from the disjointigs [57].

Finally, minimizers can also be combined with multiple data types to improve genome assembly efficiency and accuracy [122]. The assembler *ntJoin* builds an ordered minimizer sketch from both a de novo assembled genome and a reference genome [58]. This sketch forms the basis of an undirected graph where minimizers serve as nodes, and edges connect minimizers that are adjacent in at least one of the sketches. The user can assign weights to the edges, prioritizing either the de novo assembled genome or the reference genome. Following a pruning process that removes edges with low support, the sequences of minimizers along linear paths are translated into ordered oriented contigs. This approach’s strength lies in its ability to address misassembly and facilitate efficient scaffolding to a reference in an alignment-free manner.

The *Wengan* assembler employs minimizers to execute hybrid assemblies, combining short, paired-end Illumina reads with long PacBio and/or ONT reads [59]. Initially, *Wengan* constructs a cdBG from short reads, with the option for users to choose *BCALM2* for graph compaction. The presence of repeat sequences introduces branches in the dBG, potentially leading to chimeric contigs. To mitigate this, an alignment-free approach, inspired by *minimap2* (see the “Read alignment” section) [40], maps short paired-end reads to the resultant contigs. Junctions between chimeric regions exhibit lower coverage, enabling the detection and subsequent trimming or splitting of chimeric contigs into shorter, non-chimeric counterparts. In the next phase, *Wengan* capitalizes on long reads to generate synthetic paired reads, tailoring them with varying insert sizes (e.g., 0.5 kb to 200 kb with ultralong ONT reads) to span repetitive regions. These synthetic reads are then mapped to short-read contigs using the minimizer-based approach. Given their diverse insert sizes, some reads span multiple contigs, proving especially beneficial when spanning contigs containing repeats. The mapping information is instrumental in constructing a synthetic scaffolding graph, illustrating potential orientation and distances between contigs based on the mapping of synthetic reads. This graph is streamlined using information from long reads and the mapping locations of their corresponding synthetic reads to establish accurate paths, facilitating the construction and validation of the assembly backbone. Finally, contigs not encompassed in the backbone, likely associated with repeats or short sequences, are inserted by aligning minimizers in the backbone with those at the edges of the excluded contigs. This strategic employment of minimizers enables *Wengan* to achieve superior contiguity in assembling the human genome, surpassing the benchmark set by the GRCh38 reference genome.

In conclusion, the challenges of de novo genome assembly, particularly for large and complex genomes, have driven the introduction of minimizers in several steps of genome assembly. The minimizer-centric approach, exemplified by tools like *MBG*, *rust-mdBG* and *LJA*, has proven instrumental in reducing computational burden and enhancing efficiency of graph construction, significantly reducing memory requirements. Additionally, combining minimizers with diverse data types, as seen in *ntJoin* and *Wengan*, enhances assembly accuracy and efficiency. Overall, the adoption of minimizers represents a pivotal advancement in de novo genome assembly, addressing challenges associated with large genomes, computational complexity, and sequencing errors.

### Pangenomes

#### Introduction

Traditional reference genomes, which consist of linear sequences representing a single copy of each chromosome in an individual, fail to capture the genetic diversity within populations [60, 85]. This incomplete representation introduces bias in read mapping and downstream analyses, in particular “reference bias,” which is when read aligners penalize differences between the reads and the reference (i.e., genetic variations), resulting in fewer mapped reads or lower reported mapping quality [123]. Such biases impede discoveries on genotype–phenotype associations and gene function [124]. Pangenomes, encompassing the entire genomic diversity within a species or group of related species, offer a solution. Mapping sequences, particularly short reads, against a pangenome has been shown to reduce bias compared to more traditional methods (the “Read alignment” section) that map to the classic reference genome [61].

The concept of pangenomes, initially applied to bacterial genomes, has expanded to eukaryotic genomes, focusing on structural variants and haplotypes across individuals and populations. Pangenome representations range from collections of core genes and accessory genes in prokaryotes [125–127], to complex graphs of whole genomes or regions of interest capturing genetic variation in eukaryotes [60]. See review articles [128] for a history of pangenomes, [129, 130] for pangenome data structures, [131, 132] for pangenome construction, and [62, 133, 134] for their applications.

Various solutions have been proposed to store, analyze, and represent pangenomes. Originally, core and dispensable genes were identified through traditional alignment approaches like Smith-Waterman [135], which aligned linear representations of genomes or gene sets (sequence strings) of the genomes of interest. This approach distinguishes gene sets that align across all genomes (i.e., core genes in bacterial strains) from those that do not (dispensable genes) [125]. Orthology calling approaches complemented this by analyzing gene homology across genomes using tools like BLAST and OrthoMCL, categorizing genes based on their presence across gene families [136, 137]. While using genes themselves as the input streamlines this process, they depend heavily on the accuracy of the initial gene annotations [138]. However, these strategies primarily suit prokaryotic genomes, rather than eukaryotic ones. Moreover, they struggle to scale with increasing genome numbers and sizes. To address these limitations, another strategy involves indexing and compressing aligned sequences to optimize memory use and accelerate gene alignments, exploiting identical regions in sequence collections [139, 140]. This approach, though efficient in memory reduction, generally fails to adequately represent longer genetic variations, such as translocations, inversions, or duplications, due to its reliance on classical methods which assume collinearity [60, 141]. Pangenome reference graphs (PanRG) have emerged as an efficient alternative, leveraging graph structures to accurately represent genetic variation [132].

A basic approach to creating a pangenome graph involves constructing a compacted de Bruijn graph (cdBG) from a set of genomes. Recall that minimizers can be used to efficiently provide a more compact representation of such graphs (Fig. 5). However, this approach does not store information about the origin of sequences that come from different samples [99, 142, 143]. Colored cdBGs (ccdBGs) were introduced to label *k*-mers with sample information with a different color in the graph as is done in *Bifrost or Fulgor* (the “Representing de Bruijn graphs” section) [52, 97]. However, ccdBGs do not store the chromosomal coordinates, preventing the mapping of genomic features. VG (variation graph) is a toolkit for creating and manipulating pangenome graphs where each node is a sequence and paths represent potential sequences of a population. Such graph structure has been extensively leveraged by the VG team and others for DNA/RNA read alignment, variant calling, and genotyping [144]. Scaling up pangenomes to hundreds of human genomes remains computationally challenging and current efforts focus on developing methods able to accurately capture genomic variants across more genomes [131, 145].

Minimizers play a critical role in the construction and indexing of pangenome graphs, enhancing the efficiency of genome graph algorithms. These algorithms, such as *minigraph*, the *PanGenome Research Toolkit*, *Giraffe*, and *Pandora*, use minimizers, which finally results in improving their memory and time efficiency, in addition to enhancing the accuracy of read mappings [60–63].

#### Eukaryotic pangenome methods that use minimizers

A well-known tool for building pangenome graphs is *minigraph*, which is also designed for mapping sequences to the graph [60]. The *minigraph* software constructs the graph iteratively by mapping each assembled sequence to an existing graph. Nodes in the graph are sequences which are stored in the format of the reference graphical fragment assembly (rGFA) benefiting from a stable coordinate system. Such coordinates allow for referencing any sequence to the positions of an input classic linear reference genome.

To map sequences to the graph, *minigraph* adopts a strategy akin to *minimap2* (the “Read alignment” section). First, it identifies seed minimizers from node sequences and the query sequence, resulting in anchors. Then, linear chains are found without considering the graph topology. Finally, the second round of chaining takes into account whether they are connected on the graph or not. Compared to *minimap2*, minimizers can be more distant from each other in *minigraph*’s chaining allowing for mapping chromosome-long query sequences. Besides, *minigraph* is equipped with new heuristics for handling large gaps by speeding up the chaining process [60, 146]. In contrast to other graph aligners, such as *GraphAligner* and *VG* toolkit, which are limited to mapping small variations, *minigraph*’s approach allows for handling larger genomic variations. One drawback of *minigraph* is that it cannot call variations smaller than 50 bases. This limitation is addressed in *minigraph-cactus* using a base aligner [145, 147]. Furthermore, *minigraph*’s dependency on a linear reference genome for graph construction might introduce a bias, in contrast to VG. The authors contend that reference pangenomes should not replace classic linear genomes, but complement them, as reference pangenomes excel at identifying longer variants within more “problematic” regions, while linear genomes remain effective for analyzing smaller variations in more stable regions. Of note, *minigraph* has been used for constructing the first human reference pangenome [145, 147].

In contrast, there are methods that focus on smaller regions instead of whole genomes but are able to model them in different resolutions. The pangenome research toolkit (*PGR-TK*) [62] does not have a stable coordinate system but uses an index based on minimizers. It uses sparse hierarchical minimizer pairs as nodes of the graph. This framework reduces the time and storage needed to construct the graph using new parameters like the minimum distance between minimizer pairs to adjust the level of detail or variation size of interest. This has proven useful to study complex human genome regions of interest like the MHC class II locus or the ampliconic genes OPN1MW and OPN1MW2 [62].

In mapping applications, the *VG-MAP* algorithm [85], part of the VG toolkit, faces challenges with its time and cost efficiency due to the large number of paths it evaluates in the graph, being an order of magnitude slower than typical linear mappers. Conversely, *Giraffe* [61] achieves at least one order of magnitude less time than *VG-MAP* and can be even faster than linear mappers such as *BWA-MEM* (the “Read alignment” section). *Giraffe* uses several techniques to optimize the process. First, it leverages previously observed genomic paths to constrain the alignment search space, rather than combinatorially expanding the possible paths in the graph. Second, it uses a BWT to index haplotypes, split into sequences of nodes in the VG pangenome graph. Crucially, *Giraffe* uses minimizers (*k* = 29 and *w* = 11) for finding matches between reads and the node sequences, as the seed of the seed-and-extend approach. A hash table is used for indexing the minimizers where keys (*k*-mers) and values (a pointer to a sorted array of hits as graph positions) are 64 and 128 bits, respectively. Minimizers in high-scoring clusters of seeds with minimum graph distance are extended, forming gapless alignments for most low-error short reads. When gapless alignment is not possible, gapped alignment is performed using dynamic programming [61]. In summary, using minimizers not only optimizes alignment efficiency but also underscores their role in advancing pangenome mapping technologies.

#### Prokaryotic pangenome

While eukaryotic pangenome methods leverage minimizers for enhanced resolution and efficiency, prokaryotic genomes present particular challenges as well. Bacteria harbor a vast genetic diversity within a species, much of which is not captured by a single genome. The underrepresentation of genetic diversity associated with the classic linear genome references is especially problematic in bacteria. This disparity underscores the necessity of the PanRG to accurately represent the full spectrum of genetic material, especially considering that the core genes that are present in the single-reference genome are only a small percentage of the number of individual’s genes [63].

For variant calling, most graph-based methods, adept for human pangenomes, often require a linear reference genome and/or generate a genome-wide PanRG. However, *Pandora* [63] offers a novel solution capturing the diversity of prokaryotic pangenomics by introducing “local” graphs. A *pandora* PanRG is an unordered collection of several local graphs, which are directed acyclic. Each local graph is created from an MSA of a genomic region (genic or intergenic) from assemblies of different species or strains using a recursive clustering algorithm on MSA’s rows and columns. The local graphs are indexed using a minimizer scheme (with parameters of *k* and *w*) generalized to sequence graphs by considering paths of sequences with length *w* + *k* − *1* as the minimizer window. When mapping reads to PanRG, *Pandora* decides which local graph (i.e., a genomic region) is present in the sample. To do so, another graph is constructed where each node is a minimizer and an edge shows adjacent minimizers on the original local graph. *Pandora* uses a global index to map each minimizer to local graphs, which is used for comparing them to reads’ minimizers and finding hits. Finally, genotyped variants are found using a maximum likelihood approach based on a Poisson model reported in a file with variant call format where the chromosome field represents the local graph [63].

This advancement in prokaryotic genome analysis complements the progress made in eukaryotic pangenome methods, where minimizers also play a crucial role. As genome sequencing becomes increasingly widespread, pangenomes are likely to become the new standard for reference genomes. Such large amounts of data need efficient storage solutions and search algorithms. The implementation of minimizers has been key to scaling up the construction of variation graphs, with successes such as assembling the draft human pangenome [145]. The integration of minimizers across different genomic studies exemplifies their contribution to modern genomics of less well-studied species, which may exhibit even more genetic variation than humans.

### Metagenomics

Metagenomics is the study of genomic sequences from the natural environment, often in large quantities. The goal is to identify the microbial taxa that exist in complex biological and environmental samples [148, 149]. This field encounters various computational challenges in identifying samples due to the complex definition of species or subspecies of certain bacteria or viruses. The initial challenge arises from high-throughput sequencing technologies that generate millions of reads. There are two different analyses to process these large quantities of data: first, classifying the taxonomy and, second, assembling it (Table 1).

#### Metagenomics classifiers

To classify metagenomic data, a reference database is usually needed. The amount of previously stored sequences and how quickly the classifier can retrieve the data are important in determining the efficiency of the classifier [148]. Expanding the reference database can improve classification, but if the taxa are not known, or very different from the database, it can be difficult for classifiers to identify the origin of each read of the sequenced sample. Additionally, the larger the database, the longer the run time can take [148].

There are several read classifiers in the field of metagenomics that use *k*-mers and minimizers to maximize efficiency and precision. One of the first classifiers is called *MEGAN*, a metagenome analyzer that examines a set of unknown DNA sequences and compares them against databases of known sequences using BLAST [150]. *MEGAN* finds the lowest common ancestor (LCA) of BLAST hits to assign reads to taxa. This tool pre-dates the use of *k*-mers and minimizers but is an important milestone in the development of more efficient classifiers.

*Kraken* exceeds the speed and accuracy of *MEGAN* by using exact-match database queries of *k*-mers rather than the alignments of sequences [64, 151]. *Kraken*’s database contains both *k*-mers and the LCA of all organisms whose genome contains that *k*-mer. Sequences are classified by searching the database for each *k*-mer and then using the LCA taxa to determine the appropriate leaf label of a species in a phylogenetic tree with the default of *k* = 31. In short, to classify a sequence* S*, the algorithm collects all the *k-*mers within that sequence denoted as *K(S)* and then maps each *k-*mer to the LCA taxon of all the genomes that contain the specific *k-*mer. Then, the LCA taxa and the ancestors build a “classification tree” which is used to classify *S* by assigning a weight to each node calculated as the number of *k*-mers associated with it. Finally, the root to leaf path in the classification tree is scored by the sum of all the node weights within the path. The maximum score of the root to leaf path is then deemed the “classification path” and the sequence *S* is assigned to the label corresponding to its leaf [45]. One of the constraints of *Kraken* is the memory usage. The *Kraken* database requires 70 GB (based on the dataset in [65]), which can grow larger with more genomes added by the user.

*Kraken2* improved upon *Kraken* by changing the structure from a sorted pair list of (*k*-mer, LCA) indexed by minimizers to a compact hash table which is used to map minimizers to LCAs [65]. Storing only minimizers of length *s*, (*s* ≤ *k*), instead of keeping all the *k*-mers, significantly reduced the reference database to 10.6 GB based on the dataset in [65], which is a sixfold decrease in memory usage. *Kraken2* uses the standard linear-time algorithm for computing minimizers in which *s*-mers are minimizers. This algorithm uses a double-ended queue in which candidate *s*-mers are put in the back of the queue, keeping their original position in the sequence. When a new candidate is found, the old candidates with greater values in terms of lexicographical ordering are removed and the new candidate is pushed to the back of the queue [27]. The computational complexity of this approach of calculating new minimizer is O(1) in contrast to Θ(*k*) for the first version of *Kraken*.

*K2Mem* (*Kraken2* with memory) is a classifier based on *Kraken2*, bolstered with an enhanced memory requirement. The classifier detects novel minimizers from the input sequencing data and stores them to improve the classification of reads [66]. The process has two main steps. First, all reads are processed and the new minimizers are stored in an additional minimizer map revealing the taxa. Second, the same input reads are classified using the compact hash table while additional minimizers are found. Compared to *Kraken2*, *K2Mem* has better total time (from start to finish), due to the new minimizer search phase. Its classification time (time to classify a read) is similar to that of *Kraken2*, but it requires slightly more memory due to the additional minimizer map.

Most classifiers work with short reads since that is often what is available with metagenomic datasets. However, the *MetaMaps* algorithm was developed to analyze long-read metagenomic datasets. It works by mapping each long read using a minimizer-based approximate mapping strategy [67]. Since it is becoming increasingly common to have long-read datasets due to improved technology and cost efficiency, these long-read algorithms have vastly improved the field.

#### Metagenomic assemblers

The second type of software tool that we review in the field of metagenomics is metagenome assemblers (Table 1). While de novo genome assembly (the “De novo genome assembly” section) typically deals with a genome of a single species, metagenomic assemblers face two main challenges: distinguishing between repeats/orthologous sequences and species as well as coping or accounting for different coverage levels per species [152]. In this section, we survey *MetaProb2*, an algorithm that uses minimizers for assembling the metagenomic data.

*MetaProb2* is an unsupervised metagenomics binning method that uses minimizers to assemble reads into unitigs [68]. First, reads are grouped based on their common subsequence using *minimap2* (assumed to be of the same species) and then assembled using long-read de novo assembly algorithms, such as *miniasm.* The use of minimizers is critical because it stores a fraction of the *k*-mers to perform all-vs-all comparisons between sequences, which results in faster computation and lower memory usage. Based on the information provided by the overlap detection along with the paired-end reads, the assembler groups unitigs that are likely from the same species. Lastly, the inferred number of species and their abundance in the sample are kept using sequence signatures based on *k*-mer statistics. Overall, *MetaProb2* has good performance in terms of precision and recall when comparing real and simulated datasets. Recently, *metaMDBG* has been proposed [153] for metagenomics assembly from HiFi reads which works in the minimizer space (see the “De novo genome assembly” section). Minimizers are a crucial step forward in the field of metagenomic classification and assembly tools both with computational speed and memory usage.

## Minimizer alternatives

The use of minimizers has become increasingly popular in bioinformatics for efficient sequence analysis. However, several alternative methods have been proposed to increase the efficiency and overcome the limitations of minimizers, especially in scenarios with highly divergent sequences with substitutions and indels where *k*-mer-based approaches are prone to fail.

### Universal hitting sets (UHS)

Universal hitting sets (UHS) were introduced as an alternative to minimizers with the hope to decrease the resulting density. A UHS is a set of *k-*mers that is guaranteed to have at least one hit in every *L* long sequence. While a complete set of all possible* k*-mers serves as a UHS, the focus lies in finding the optimal UHS, the smallest set satisfying this criterion [36].

The process of identifying the most compact UHS presents a significant challenge, classified as nondeterministic polynomial-time (NP) hard. However, certain heuristic approaches offer partial solutions [36, 154]. The *DOCKS* algorithm is one such heuristic, operating in a two-phase manner: initially, it constructs a complete dBG and determines the minimum number of vertices required to remove to make the dBG acyclic. Subsequently, *DOCKS* eliminates the smallest possible vertex set to ensure that it covers all paths of length (*L-k*). Although the initial phase is polynomially solvable, the latter phase is NP-hard necessitating heuristic strategies for better resolution [36]*. PASHA* [154] is a method similar to *DOCKS*, which is identical to *DOCKS* in its first step but uses a randomized parallel algorithm to enhance speed and efficiency. While UHS provides a smaller and more evenly distributed set than a minimizer scheme, its computational demand escalates exponentially with an increase in *k*, limiting *DOCKS* and *PASHA*’*s* practical application to *k* < 13 and *k* < 16, respectively. Nonetheless, these methods can reduce the density by up to 30% compared to random minimizers [155].

Most of the algorithms designed for constructing a UHS offer the flexibility to take a target sequence as input. This enables the algorithms to incorporate *k*-mers from this target sequence into the final UHS with a higher probability than *k*-mers that are not in the target sequence. This feature is particularly beneficial for tailoring the UHS to be more effective for sequences of interest, such as the human genome. In the subsequent section, we will explore polar sets, a closely related concept that addresses the challenge of creating sequence-specific sketches.

### Sequence-specific minimizers via polar sets

Unlike UHS, which ensure coverage by guaranteeing at least one hit in every *L*-long sequence, polar sets are designed to guarantee dispersion. This means that each pair of selected *k*-mers in a polar set is spaced at least *L* nucleotides apart, ensuring that each *L*-long window is hit at most once [156]. Polar sets achieve a low-density sketch, approximating the theoretical lower bound (1/*w*), and are effective even with large *k* values. Finding polar sets is shown to be NP-hard, but a heuristic algorithm is proposed to identify their approximations in linear time. Similar to UHS, polar sets require a lookup table for each *k*-mer in the query sequence. This is a drawback for non-random minimizers that do not use a hash function [156].

### Asymptotically optimal minimizers

*Miniception* introduces an innovative approach by utilizing a secondary smaller minimizer to improve the efficiency of the primary, larger minimizer [32]. Specifically, the “smaller” refers to a minimizer with a smaller window size (*w*_*0*_) and *k*-mer length (*k*_*0*_), whereas the “larger” minimizer operates with a larger window size (*w*) and *k*-mer length (*k*), where *k* = *k*_*0*_ + *w*_*0*_ and *w* > *w*_*0*_. This dual-minimizer setup has been shown to achieve an upper bound expected density of 1.67/(*w* + 1), which is lower than the 2/(*w* + 1) density of traditional random minimizers. Moreover, similar to the random minimizer, *Miniception* operates with linear time complexity, making it more efficient than UHS or polar sets, which are slowed down by their need for table lookups. Such *k*-mer precomputation of a lookup table during sketching is not a requirement for *Miniception*, allowing high scalability to large values of *k* without the overhead of managing precomputed *k*-mer sets. While having these advantages in time and memory performance, the lower bound of the resulting sketch (1.67/(*w* + 1)) is higher than the theoretical lower bound (1/*w*), which can be achieved using UHS or Polar Sets.

### Syncmers

Minimizers are a context-dependent method, meaning that the selection of a *k*-mer can be influenced by mutations in positions outside of the *k*-mer within the same window. Syncmers are designed with the principle that resistance to mutation (i.e., degree of conservation) is more important than achieving a sketch with low density. Syncmers work by selecting *k*-mers by inspecting the position of the smallest-valued substring of length *s* (*s*-mer where *s* < *k*) within the *k*-mer [23]. Variations of syncmers have been proposed, one of which is closed-syncmer; a *k*-mer is selected if the smallest *s*-mer is located at either its first or last position, making syncmers a context-free method. Selection of a k-mer solely depends on its own sequence, not on its flanking sequence. The authors also present evidence that syncmers can attain higher conservation and lower density compared to minimizers, as utilized by the *minimap2* read mapper and the *Kraken* taxonomy classification algorithm [23]. It is shown theoretically that syncmers can decrease the chaining time without significantly increasing extension time in a seed-chain-extend heuristic of read alignment [157].

### Strobemers

The syncmer scheme represents an advancement of *k*-mer-based methods by minimizing the impact of mutations through a context-free selection process. However, they are, at their core, still susceptible to the intrinsic limitations of *k*-mer-based approaches, where even minor mutations can alter the selection and representation of *k*-mers, potentially affecting alignment accuracy and efficiency. In contrast, strobemers aim to address and mitigate these limitations more effectively by employing a novel strategy that links two or more spaced *k*-mers (strobes). These strobes are extracted from non-contiguous sequences (variable intervals within the sequence), resulting in a higher level of flexibility and robustness achieved by strobemers [158]. This approach allows for the accommodation of indels and more complex mutations without losing the ability to accurately identify and align sequences [158]. Despite the advantages of strobemers, they do require more parameters to optimize than other techniques like minimizers.

Building on the concepts of strobemers and syncmers, *Strobealign* is designed as a faster and more accurate alternative to traditional aligners (e.g.,* bowtie2*,* minimap2*) for aligning read sequences. *Strobealign* works by first using syncmers to create a sketch of the sequence. These sketches are then linked to form strobemers, employing variable size and fuzzy seeds for alignment. This innovative process reduces the number of seed candidates, resulting in enhanced speed maintaining high accuracy [159].

In summary, alternative methods to minimizer enhance sequence sketching by lowering the density and improving the resilience to mutations. Each of these methods offers a unique approach yet, retaining similarities to minimizers. These methods present varied trade-offs between density, speed, scalability, and complexity of parameters to choose, making the choice of the most suitable approach dependent on the specific needs and requirements of the analysis.

## Discussion and conclusion

Minimizers are an effective approach to reduce the data complexity and volume that needs to be dealt with by genomics methods to efficiently utilize or simply query information. This is achieved by creating “sketches” of sequences that occupy less space compared to the sequences themselves. We presented examples of extensions of the minimizer scheme and applications in different data processing techniques. The versatility and effectiveness of minimizers make them a valuable tool for solving a wide range of problems, particularly in genomics. Here, we reviewed five important applications of minimizers including read alignment, read correction, genome assembly, pangenomics, and metagenomics. Specifically, when it comes to de novo genome assembly, the combination of de Bruijn graphs and minimizers is a powerful approach, achieving contiguous, high-quality assemblies of large genomes. In metagenomics, minimizers significantly improved classification and assembly methods tackling many challenges of complexity that arise with millions of short-reads and the memory space used by genomic sequences. Nevertheless, minimizers have several other uses such as *k-*mer counting [160], sequence compression [161], contamination detection and sequence classification [162, 163], querying databases [164, 165], synteny detection [166] in addition to variant calling, and multiple sequence alignments [167].

Limitations of minimizers, especially in reducing density to the theoretical minimum, inspired researchers to devise alternative algorithms such as universal hitting sets (UHS), syncmers, and strobemers. Another limitation of minimizers became evident in estimating sequence similarity which is shown to be a biased estimator [168] which is addressed by developing “minmer,” a new scheme where several *k*-mers are selected per window [169]. While these new algorithms have their own constraints, they attempt to enhance the efficiency of minimizers in specific scenarios and to a certain extent. Another challenge is the choice of parameters including the *k* value and the window size, which is not the case for full-text indexing approaches like FM-index [140] or MOVI [170]. Notably, a variable-length minimizer scheme (called finimizers) has recently been proposed guaranteeing maximum minimizer frequencies [171]. Overall, minimizer-based approaches will continue to evolve and improve as technology advances and the cost of sequencing and memory usage decreases. Specifically, lines of research are concerned with theoretical error analysis and investigating how to choose the efficient minimizer ordering to best approach the theoretical minimum density.

The minimizer and alternative approaches can be used in several new applications, specifically for methods that are based on *k*-mer counting (metagenomics abundance estimation), sequence comparison (gene clustering), and feature selection (convolutional neural networks [172, 173] and gene regulatory network [174, 175]), in addition to preprocessing techniques, such as partitioning sequence data for efficient parallel processing and storage.

## Supplementary Information

Additional file 1. Review history.

## Data Availability

Not applicable.
