# Supplementary material for: When less is more: sketching with minimizers in genomics
Source: Genome Biol. 2024 Oct 14;25:270. doi: 10.1186/s13059-024-03414-4 (PMC11472564; doi:10.1186/s13059-024-03414-4)
Supplement: Supplementary file 1 — Additional file 1. Review history. [file 13059_2024_3414_MOESM1_ESM.docx]

# **1^st^ round**

**Reviewer 1**

This manuscript proposes a review of sketching methods, across many methods within bioinformatics, focusing particularly on the use of minimizers. Although this review is valuable, there are many parts that are superficial and not so technically accurate e.g. on hashing, alignment, and in many places in the applications.

Here are some examples of how it could be improved at some places:

1. "Hashing, a computer science technique, transforms any given string (a k-mer in our case) into an index of bit strings" This paragraph needs to be revised entirely. This is not the right definition of hashing, also, there is no "index" here. It is better that you only explain hash functions here, no need to go into data structures.

2. The paragraph "While the first aligners [..]" is misleading. It only selects the BWA suite and NGMLR somewhat arbitrarily, omitting the rich history of short read and long read aligners, e.g. Bowtie, blasr, etc. If you keep this section, please make it more complete, possibly also cite https://genomebiology.biomedcentral.com/articles/10.1186/s13059-023-02972-3.

3. Regarding the sentence: "[..] the best paths are chosen [..]" This is a speculation, and a vague one at that.

This is by no means an exhaustive list. I would recommend that senior authors proofread the manuscript, focusing on computational aspects, and also the applications section (Section 3). I would be happy to look at a revised version of the manuscript.

**Authors’ response**

Reviewer #1

This manuscript proposes a review of sketching methods, across many methods within bioinformatics, focusing particularly on the use of minimizers. Although this review is valuable, there are many parts that are superficial and not so technically accurate e.g. on hashing, alignment, and in many places in the applications.

RESPONSE: Thanks for considering this work valuable. Our focus in this manuscript is to provide a survey and high level idea of minimizer for the broader community of bioinformaticians, biologists and geneticists. To address the comments, we have improved the whole manuscript by clarifying the concepts and adding more algorithmic details.

Here are some examples of how it could be improved at some places:

1. "Hashing, a computer science technique, transforms any given string (a k-mer in our case) into an index of bit strings" This paragraph needs to be revised entirely. This is not the right definition of hashing, also, there is no "index" here. It is better that you only explain hash functions here, no need to go into data structures.

RESPONSE: We have revised the section completely. Now we do not mention the indexing and focus on the hashing function.

2. The paragraph "While the first aligners [..]" is misleading. It only selects the BWA suite and NGMLR somewhat arbitrarily, omitting the rich history of short read and long read aligners, e.g. Bowtie, blasr, etc. If you keep this section, please make it more complete, possibly also cite

https://genomebiology.biomedcentral.com/articles/10.1186/s13059-023-02972-3.

RESPONSE: Thanks for pointing this out. We did not plan to describe the history of read aligners and our goal was to describe a few representative tools in the field. Now we revised the section and mentioned BWA, Bowtie, and BLASR. We also cited the suggested review paper which we found comprehensive. We also mention that the focus of the manuscript is to describe those read aligners that benefit from minimizers.

3. Regarding the sentence: "[..] the best paths are chosen [..]" This is a speculation, and a vague one at that.

RESPONSE: We have updated this paragraph by adding more details on how the algorithm works.

This is by no means an exhaustive list. I would recommend that senior authors proofread the manuscript, focusing on computational aspects, and also the applications section (Section 3). I would be happy to look at a revised version of the manuscript.

RESPONSE: We did our best to revise the whole manuscript and fact check all the statements. We appreciate providing us the opportunity to improve our work.

**2^nd^ round**

**Reviewer 1**

The authors have submitted a revised manuscript for this review article. Many of the issues and inaccuracies I had spotted in the first manuscript have been corrected, but overall many issues are still present. I am reporting them in the following comments.


The article has been improved, but at times is still below standard for publication. In particular the alignment section needs work due to numerous imprecisions, as reflected by my comments.


Overall, the breadth of the applications reviewed make a strong case on the contribution of minimizers to bioinformatics.


1. Table 1: MBG description is incorrect: it is not a minimizer-space DBG, it uses minimizers but it operates in base-space. Minimizer-space is when the alphabet itself consists of minimizers and the bases are forgotten (which is the case in rust-mdBG but not MBG).
2. “dividing the sequence into smaller, manageable substrings.” This is a minor semantic suggestion: I wouldn’t use the term “dividing”, which implies partitioning, but here windows are self-overlapping.
3. “[..] compact representations, or sketches, of sequences which occupy a “minimized” space in comparison to the sequences themselves.” This statement is somewhat technically true but can induce much confusion. The “minimize” part of minimizers isn’t about storing a sequence in minimal space, but about finding minimal k-mers within a window. Another source of confusion is the “compact representation” part which implies that a minimizer representation somehow preserves all the information of the original sequence, which is not true. I would advise against using “compact representation” but instead e.g. “approximation” to convey that there is information loss.
4. “A useful minimizer scheme must have two properties.” I do not believe these properties are necessary, rather, they are consequences. This should be reworded. It looks like these “properties” are actually corollaries/consequences of using a minimizer scheme.
5. In the revised hashing section, the following new sentence still needs to be revised for accuracy “a fixed-sized value, which reduces the memory requirements as the function can be selected to output numbers smaller than k.”: a hash function does not necessarily save space (it can be bijective), and “numbers smaller than k” is an error, I suppose 4^k was meant instead.
6. “De novo genome assembly deals with the problem of reconstructing a consensus sequence of length G“ Not really, this is a simplification from the pre-diploid assembly era. What about multiple chromosomes, and each having 2 (or more) haplotypes?
7. “Some sketching examples are locality-sensitive hashing, compressed string indexes or bloom indexes [22]. “ Is the middle one really a sketching method? All bases are kept (even in compressed form), hence this does not appear to be sketching anymore.
8. “However, this efficiency decreases as k increases” this is not always true. To give two anecdotes, see e.g. GGCAT where longer k-mers actually lead to faster execution; MEGAHIT also has memory usage largely independent from the chosen k-mer size.
9. Caption Figure 2: “For the sequence 1 with w=8, considering four windows, selected minimizers are ACT at positions 2 and 7“ This appears incorrect: with w=8 there are only 2 windows, and perhaps sequence 2 is meant.
10. “using functions to assign bit strings (numerical values in binary format)” why not just say “numerical values”?
11. Figure 3: caption is incorrect with respect to red box showing minimal value.
12. The formulation “but adding a term resulted in” is awkward.
13. The sentence “the reference genome is vast, usually with many unresolved regions” is problematic. The entire paragraph needs to be revised by a senior author.
14. The sentence “A wrongly placed or misaligned read often leads to a falsely-identified variant” needs a source.
15. In the sentence "taking computation close to linear in the length of the reference in average case", why is the minimizer computation not strictly linear-time in the worst case with Karp-Rabin hashing?
16. For the formulation “using a dynamic approach” did you mean dynamic programming?
17. The sentence “to maximize matching bases between anchors considering cost functions of gap length (a combination of affine and/or log functions [36,37])” is convoluted and potentially inaccurate: why is it “and/or”?
18. For the sentence “allow the use of dynamic programming for shorter chunks of sequences” this is unrelated to minimizers, but rather a consequence of using seeding.
19. The sentence “Since minimizers from repetitive regions are sampled with a higher frequency, which in turn falsely increases seed hits.“ is not grammatically correct.
20. Typo/grammar: “for a better reflecting biological variation”, “leading to different penalizing of opening or extending a gap”
21. The sentence “As computations of the Jaccard [..]” is a repeat of the previous sentence.
22. The concept of anchors, chains, and chaining, keep being redefined on the descriptions of all mapping tools. This would be better factorized into a separate paragraph and re-used appropriately in tool description, with only the description of conceptual changes to the canonical definition.
23. “Compared to BWA-MEM and minimap2, Chromap performed similarly on 100 and 150 bp paired-end data with 98% accuracy on simulated data”. This can be reworded more accurately to state that BWA-mem, minimap2, and chromap, all had 98% accuracy on this data.
24. “Finally, all paths with low support (i.e., representing rare k-mers in the read dataset) are collapsed because
they probably originate from sequencing errors or polymorphisms” for polymorphism, this is not related to low support, but rather to the bubble topology.
25. In the first paragraph of 3.3, OLC assembly is painted in an unfairly negative and possibly simplistic light. How about the use of minimizers in hifiasm, a competitive OLC assembler?
26. “graph can be traversed by an Eulerian walk” this is in practice not performed. Contigs are just the simple paths of the simplified dBG. There is no Eulerian walk traversal.
27. “The number of unique k-mers and the possible overlaps between them is independent of sequencing depth” This is only true after a certain sequencing depth threshold, and even so, it is an exaggeration to state that it’s fully independent; but it can be stated that the number of nodes of error-cleaned dBGs plateau at higher sequencing depths (but in uncleaned dBGs, sequencing errors accumulate and generate more erroneous nodes). Also the overlaps are fully determined by the set of k-mers it, so there is never variability on the set of possible overlaps.
28. “to assemble a human genome in >4Mb contigs” this must be a typo, not Mb but million.
29. Figure 4 typos: “MGB”, “rust-mdGB”. But otherwise, this is a good figure.
30. “Subsequently, a dBG is constructed in the minimizer space” [..] “ Following the minimizer-space dBG construction”. No: if the alphabet is nucleotides, this is not a minimizer-space dBG but a regular nucleotide-space dBG. You can instead write that “a dBG is constructed with minimizers as nodes”.
31. In BCALM2 description: “Here, the parameter l is chosen to be less than k, ensuring that each k-mer possesses a minimizer at both its prefix and suffix”. This is not technically correct. There is nothing special about l, which follows the standard minimizer definition. The only novelty here is to make sure to select minimizers in k-1 prefixes of k-mers as well as select in k-1 suffixes of k-mers.
32. “Additionally, k-mers with neighboring hashing of minimizers” is vague and “Since k-mers in different same BBF blocks” has an extra word.
33. “However, none of these tools could scale up to many human genomes.” They can, with challenges, as per https://genomebiology.biomedcentral.com/articles/10.1186/s13059-023-03098-2
34. “It cannot call structural variations smaller than 50 bases” Neither can it call any variation (eg SNPs) smaller than 50 bases, not just structural.
35. “Kraken2 determines which l-mers are minimizers by using a sliding window minimum algorithm.” Isn’t this the standard linear-time algorithm for computing minimizers? This is not exclusive to Kraken2, most minimizer-based tools use it.
36. “we survey two algorithms that use minimizers for assembling the metagenomic data, namely Global clustering algorithm and MetaProb2.” The first program is not an assembly program and should probably not be included in this section.
37. "First, reads are grouped based on their overlap that they share a common subsequence using minimizers (assumed to be of the same species) and then assembled using miniasm (de novo assembly tool) and minimap2 (the mapping tool; Section 3.1)," This sentence needs to be revised for technical accuracy, at two locations: the overlap part, and minimap2 is not an assembler.
38. “with highly divergent sequences with substitutions and indels where k-mer based approaches are susceptible” Unsure here is susceptible is the right adjective. Did you mean, prone to failure?
39. In the conclusion, “This is achieved by representing sequences with shorter subsequences” is vague, as sequences are not transformed into an equivalent representation, but rather sketched into a potentially highly lossy representation.
40. In the conclusion, “from combining insights of algorithmic functions” has unclear meaning.
41. In the conclusion, “Minimizers are used to process data in a way that is efficient and suitable for numerical operations. These techniques have certain trade-offs and limitations such as probabilistic approximate results and hash functions with the potential for collisions.” is vague and appears to confuse multiple independent concepts, as minimizers do not have to be combined with hashing.

**Reviewer 2**

This is a well-written review focused on minimizers, with a reasonably substantial discussion of k-mers. I learned some new things from it, which was nice!

(I am aware I am coming in as a second round reviewer!)

I think this is already quite good, but could be improved by adding some specific content above section 3. In brief, I found the transition between section 2 (here's what minimizers are) and section 3 (here's how they're used) to be pretty abrupt. From a teaching/learning perspective, the transition could be improved with a simple example and a diagram - the minimap example seems just about perfect for this!

I would also welcome a little bit more explanation of how minimizers differ from using all the k-mers. I can intuit a lot of it from the discussion, but a simple summary statement would be useful. It's entirely possible it's there and I missed it.

**Reviewer 3**

Overall assessment
------------------

The manuscript is a survey about minimizers' applications in genomics. Minimizers are sketching algorithms that retain a (typically, small) sample of the kmers from a sequence S, hence permitting to save considerable memory and computing time. They found several important applications, as described in the manuscript.

In my opinion the survey offers a quick start into the concept of minimizers and overview some important applications. It is not, however, particularly technical but I would not regard this as a problem given that it is useful to also give general overviews without too much technicalities that can prevent from getting the core intuitions.

Thus, I'm in favour of accepting this manuscript *provided that* the authors include some changes that I list below.

Major
-----
First, there are some corrections to be made (see "Minor", below).
Second, I suggest another important application of minimizers, that is:
representing de Bruijn graphs (dBGs). This should be definitely added to Table 1. Currently, the manuscript talks about dBGs when discussing de novo assembly in Section 3.3 and it seems to mix the two concepts of "assembly" and "dBG construction". These two things are related, sure, but they are not the same. A dBG can help the process of de novo assembly but it is not the only approach. So, I would suggest to distinguish between the two and to have a separate Section -- before 3.3 -- headed "Representing de Bruijn graphs". As you mention, dBGs are fundamental for many applications in genomics.

This section should include recent development made in using minimizers to compactly represent dBGs and colored dBGs.
Currently, you only mention Bifrost which is not the state of the art anymore.
Another approach based on minimizers is Blight [6], although the current state of the art is an approach called SSHash [1,2]. SSHash represents a (possibly, weighted) de Bruijn graph using random minimizers and minimal perfect hashing. It has also been used to build positional indexes over collection of references [3], and to represent colored compacted de Bruijn graphs [4,5]. For example, Fulgor [4,5] is a colored de Bruijn graph index based on minimizers that solves the problem of read (pseudo) alignment, hence could also be mentioned in Section 3.1.

Minor
-----
 - Abstract: "minimizers, allows" --> "minimizers allow"

- Figure 1: what is the order being used here? Lexicographic? Then the orange kmer selected should not be "GAC" but "ACC".

- Section 2.3, when you talk about orders, I would mention (if not done already) that in practice we use a pseudo-random hash function to implement the order (hence, it is essentially random) because it performs better than lexicographic order and allows to compare the hashes of the kmers rather than the actual strings themselves.

- caption of Figure 2: "(≥w+k-1),it..." --> "(≥w+k-1), it..." (missing blank space).

- Section 2.4: "A hashing function" --> "a hash function"
"which reduces the memory ... output numbers smaller than k." First, I do not get what you mean; please reformulate. Second, perhaps you meant "smaller than |\Sigma|^k".

- At the end of Section 2.4, there are problems with the base of the logarithms.
It is not \Sigma, but |\Sigma|, i.e. the size of the alphabet (e.g., 4 for DNA).
Also, the Big-Oh term is not O(1/w) but o(1/w), that is a *little-oh* not a Big-Oh.
This makes a large difference, both in theory and practice.

- When you talk about BCALM2, you should definitely also mention GGCAT [7] which is a substantial improvement over BCALM2 (much faster in practice).

- Please, use a pageref when referring to Table 1.

- Section 4.2: "Finding polar set is shown to be NP-hard but a heuristic algorithm is proposed to identify them in linear time." -- This sentence is contradictory:
the problem is NP-hard, meaning no polynomial-time algorithm exist (assuming P is not equal to NP), hence you can only find an *approximation* to polar sets.

- Section 4.3: at the end of the section you should mention that while Miniception does not require the storage of a precomputed set, sequence-specific schemes can essentially reach the lower bound of 1/w in density whereas Miniception cannot.

- Section 5: "However, as algorithms and computational hardware...less significant."
First, I do not understand why reference [33] is cited. It makes no sense to me.
Second, I would not dare to make such a statement. Computational improvements are far more likely to be coming from algorithmic design and not from hardware improvement.

- Section 5: the last sentence ("Other lines of research...") logically belongs to the previous paragraph where you talk about efforts spent in reducing the density of minimizers.


References
----------

[1] Sparse and Skew Hashing of k-mers, Giulio Ermanno Pibiri, Bioinformatics, 2022

[2] On Weighted k-mer Dictionaries, Giulio Ermanno Pibiri, Algorithms for Molecular Biology, 2023

[3] Spectrum preserving tilings enable sparse and modular reference indexing, Fan et al., In 27-th International Conference on Research in Computational Molecular Biology, 2023

[4] Fulgor: A fast and compact k-mer index for large-scale matching and color queries
Fan et al., Algorithms for Molecular Biology, 2024

[5] Meta-colored compacted de Bruijn graphs, Pibiri et al., In 28-th International Conference on Research in Computational Molecular Biology, 2024

[6] BLight: Efficient exact associative structure for k-mers, Marchet et al., Bioinformatics, 2021

[7] Extremely fast construction and querying of compacted and colored de Bruijn graphs with GGCAT, Cracco, Andrea, and Alexandru I. Tomescu, Genome Research, 2023

**Authors’ response**

# Reviewer #1:

The authors have submitted a revised manuscript for this review article. Many of the issues and inaccuracies I had spotted in the first manuscript have been corrected, but overall many issues are still present. I am reporting them in the following comments. The article has been improved, but at times is still below standard for publication. In particular the alignment section needs work due to numerous imprecisions, as reflected by my comments. Overall, the breadth of the applications reviewed make a strong case on the contribution of minimizers to bioinformatics.

**RESPONSE**: We thank the reviewer for the positive assessment of our work. We appreciate the detailed and comprehensive comments, which allowed us to improve the manuscript further.

1. Table 1: MBG description is incorrect: it is not a minimizer-space DBG, it uses minimizers but it operates in base-space. Minimizer-space is when the alphabet itself consists of minimizers and the bases are forgotten (which is the case in rust-mdBG but not MBG).

**RESPONSE**: Thank you for pointing out this. We corrected the description of MBG in Table 1. Now it is described as a “minimizer-based dBG”.

2. “dividing the sequence into smaller, manageable substrings.” This is a minor semantic suggestion: I wouldn’t use the term “dividing”, which implies partitioning, but here windows are self-overlapping.

**RESPONSE**: We have reworded the sentence for a more accurate introduction, now it reads:

K-mers are useful for analyzing large DNA or RNA sequences, as they allow storing and manipulating these sequences using smaller, more manageable substrings.

3. “[..] compact representations, or sketches, of sequences which occupy a “minimized” space in comparison to the sequences themselves.” This statement is somewhat technically true but can induce much confusion. The “minimize” part of minimizers isn’t about storing a sequence in minimal space, but about finding minimal k-mers within a window. Another source of confusion is the “compact representation” part which implies that a minimizer representation somehow preserves all the information of the original sequence, which is not true. I would advise against using “compact representation” but instead e.g. “approximation” to convey that there is information loss.

**RESPONSE**: We have changed the wording to avoid possible confusion. Now it reads:

The minimizers scheme is a sequence analysis approach to create approximate representations of sequences, or sketches, which occupy a reduced space in comparison to the sequences themselves.

4. “A useful minimizer scheme must have two properties.” I do not believe these properties are necessary, rather, they are consequences. This should be reworded. It looks like these “properties” are actually corollaries/consequences of using a minimizer scheme.

**RESPONSE**: Thank you for this important distinction. We have now clarified this in the sentence:

A minimizer scheme has two important consequent properties.

5. In the revised hashing section, the following new sentence still needs to be revised for accuracy “a fixed-sized value, which reduces the memory requirements as the function can be selected to output numbers smaller than k.”: a hash function does not necessarily save space (it can be bijective), and “numbers smaller than k” is an error, I suppose 4^k was meant instead.

**RESPONSE**: Thank you for pointing out this potential source of confusion. The benefit of using hash functions in terms of memory efficiency lies in reducing the size of the stored values. We did not consider the number of hash values that are stored in a hash table (which could be compared to 4^k^) as was suggested in the first round of revision. We have corrected the wording as:

A hash function transforms a given string of arbitrary size (*k-*mers in our case) into a fixed-sized value. In doing so, the resulting representation will typically occupy less space [29] (e.g., hashing into 32-bit values [30] results in space saving for genomic k-mers for k>16 assuming a 2-bit encoding per nucleotide).

6. “De novo genome assembly deals with the problem of reconstructing a consensus sequence of length G” Not really, this is a simplification from the pre-diploid assembly era. What about multiple chromosomes, and each having 2 (or more) haplotypes?

**RESPONSE**: Indeed, there is a line of research on phased/haplotype-resolved assembly. To make the paragraph clearer, we added the following sentence:

This problem has also been extended to assemble all haplotypes of a diploid or polyploid species.

7. “Some sketching examples are locality-sensitive hashing, compressed string indexes or bloom indexes [22].” Is the middle one really a sketching method? All bases are kept (even in compressed form), hence this does not appear to be sketching anymore.

**RESPONSE**: Thank you for this correction. We changed the sentence so it now reads:

Some sketching examples are locality-sensitive hashing, minimizers or bloom indexes.

8. “However, this efficiency decreases as k increases” this is not always true. To give two anecdotes, see e.g. GGCAT where longer k-mers actually lead to faster execution; MEGAHIT also has memory usage largely independent from the chosen k-mer size.

**RESPONSE**: We thank the reviewer for pointing this out. We have adapted the paragraph removing the problematic wording and provided a more nuanced description:

Note that the number of possible unique k-mers increases exponentially with k, quickly exceeding |S|. However, in practice, the number of observed k-mers is bounded by |S|-k+1 and many k-mers are typically repeated [3]. As a result, typical k values are kept within the range of 20 to 200 and each distinct observed k-mer is stored only once, along with their frequencies and/or their positions, depending on the application.

9. Caption Figure 2: “For the sequence 1 with w=8, considering four windows, selected minimizers are ACT at positions 2 and 7“ This appears incorrect: with w=8 there are only 2 windows, and perhaps sequence 2 is meant.

**RESPONSE**: Thank you for pointing out this mistake. We have corrected the caption:

For sequence 1, using w=6, the selected minimizer in the first window (partly covering the underlined exact match) is ACT starting at position 2.

10. “using functions to assign bit strings (numerical values in binary format)” why not just say “numerical values”?

**RESPONSE**: Thank you for your suggestion. We have simplified the sentence as:

An alternative to lexicographic ordering is using functions to assign numerical values to *k*-mers.

11. Figure 3: caption is incorrect with respect to red box showing minimal value.

**RESPONSE**: We are sorry for the mistake. The sentence was left unchanged from the previous version of the manuscript where there had been a red box around the minimum hash value. We revised the caption:

The *k*-mer with the lowest hash value in a window is selected as the minimizer.

12. The formulation “but adding a term resulted in” is awkward.

**RESPONSE**: we revised the text and removed this phrase.

Zheng et al. provided explicit conditions only on k (i.e., k≥(3+ε)log|Σ|(w+1)) for the expected density of 2/(w+1)+o(1/w) [29].

13. The sentence “the reference genome is vast, usually with many unresolved regions” is problematic. The entire paragraph needs to be revised by a senior author.

**RESPONSE**: We have revised the paragraph and it now reads:

Read alignment involves placing and comparing DNA or RNA sequencing reads to a reference genome or transcriptome. The goal is to identify the best match between a given read and the reference, since this is the best hypothesis for where the read originated. A naive approach to read alignment would be to use brute force by checking all possible positions, but this is impractical due to the vast number of reads generated by sequencing technologies. Additionally, read alignment can be computationally demanding because the reference genome may contain repeated sequences where a read can map to with equal probability. Furthermore, sequencing errors and true genetic variability within a sample can introduce differences between the read and its matching location within the reference.

14. The sentence “A wrongly placed or misaligned read often leads to a falsely-identified variant” needs a source.

**RESPONSE**: We have added two references for this statement.

Alser, M., Rotman, J., Deshpande, D. et al. Technology dictates algorithms: recent developments in read alignment. Genome Biol 22, 249 (2021).

Olson, N.D., Wagner, J., Dwarshuis, N. et al. Variant calling and benchmarking in an era of complete human genome sequences. Nat Rev Genet 24, 464–483 (2023).

15. In the sentence "taking computation close to linear in the length of the reference in average case", why is the minimizer computation not strictly linear-time in the worst case with Karp-Rabin hashing?

**RESPONSE**: Thank you for bringing this to our attention. We wanted to distinguish between O(*w.|s|*) and O(|*s*|), for a sequence *s* with length *|s|,* so that the latter is achieved in the average case with minimap’s implementation (<https://doi.org/10.1093/bioinformatics/btw152>) (Other implementations also exist <https://curiouscoding.nl/posts/fast-minimizers/>). However, it is correct that the minimizer algorithm, when implemented with a monotonic queue and the rolling hash algorithm, is strictly linear-time in |s|. We have updated the text to reflect this and to avoid any confusion.

…*Minimap2* first finds minimizers within the reference, with computation time being linear in terms of the length of the reference.

16. For the formulation “using a dynamic approach” did you mean dynamic programming?

**RESPONSE**: Yes. We meant dynamic programming. We clarified it in the manuscript:

Then, a chain is formed from a set of collinear anchors using a dynamic programming approach.

17. The sentence “to maximize matching bases between anchors considering cost functions of gap length (a combination of affine and/or log functions [36,37])” is convoluted and potentially inaccurate: why is it “and/or”?

**RESPONSE**: The original minimap2 (doi:10.1093/bioinformatics/bty191, section 2.2.1) considers a 2-piece affine gap cost (which we had described it by “combination of affine functions”). On the other hand, the improved version (doi:10.1093/bioinformatics/btab705, section 2.3) uses the following function.

Considering d as constant, the cost function is an affine function in the number of gaps (*G/2d*) and logarithm function in gap length log(1+g_i_), (we aimed to describe this as an affine and log function in gaps). Thus, the old and new approaches of minimap2 are mentioned using “**and/or**”. To avoid inaccuracies in the high-level description of the algorithm in the manuscript, we revised the text as follows. We also provided two references so that interested readers can check the details.

Then, a chain is formed from a set of collinear anchors using a dynamic programming approach to maximize matching bases between anchors considering a customized cost function of gap length [36,37].

18. For the sentence “allow the use of dynamic programming for shorter chunks of sequences” this is unrelated to minimizers, but rather a consequence of using seeding.

**RESPONSE**: Thanks. We have removed this sentence.

Minimizers here help to avoid an exhaustive per-base search of seed matches.

19. The sentence “Since minimizers from repetitive regions are sampled with a higher frequency, which in turn falsely increases seed hits.“ is not grammatically correct.

**RESPONSE**: This has been corrected and now reads:

Building on top of minimap2, Winnowmap introduced a weighted-minimizer sampling algorithm [54]. Minimap2 ignores frequent minimizers because minimizers from repetitive regions are sampled more often, which artificially increases seed hits.

20. Typo/grammar: “for a better reflecting biological variation”, “leading to different penalizing of opening or extending a gap”

**RESPONSE**: This has been revised and now reads:

It tries to find the solution to seed chaining with a concave gap function to differently penalize opening or extending a gap.

21. The sentence “As computations of the Jaccard [..]” is a repeat of the previous sentence.

**RESPONSE**: We revised the text as follows:

Due to the expensive computational costs for comparing a read and the reference sequence, *MashMap* uses *MinHash* on minimizers rather than all *k*-mers.

22. The concept of anchors, chains, and chaining, keep being redefined on the descriptions of all mapping tools. This would be better factorized into a separate paragraph and re-used appropriately in the tool description, with only the description of conceptual changes to the canonical definition.

**RESPONSE**: That’s a very good suggestion. Anchors and chains are described at the beginning of the section, where *minimap2* is described. We removed redundancy in other paragraphs. We also added Figure 4, a simplified schematic of the *minimap2* algorithm to clarify this (suggested by the other reviewer).

23. “Compared to BWA-MEM and minimap2, Chromap performed similarly on 100 and 150 bp paired-end data with 98% accuracy on simulated data”. This can be reworded more accurately to state that BWA-mem, minimap2, and chromap, all had 98% accuracy on this data.

**RESPONSE**: We revised the text as follows:

*BWA-MEM, minimap2,* and *Chromap* all performed similarly, with 98% accuracy for simulated 100 and 150-bp paired-end data.

24. “Finally, all paths with low support (i.e., representing rare k-mers in the read dataset) are collapsed because they probably originate from sequencing errors or polymorphisms” for polymorphism, this is not related to low support, but rather to the bubble topology.

**RESPONSE**: We expanded on this sentence to make it more accurate, now it reads:

Finally, all paths with low support (*i.e.,* representing rare k-mers in the read dataset) are pruned because they probably originate from sequencing errors. Moreover, bubbles in the graph, often caused by polymorphisms or repeated regions, may be collapsed based on criteria like read coverage and path length, depending on the specific dBG implementations.

25. In the first paragraph of 3.3, OLC assembly is painted in an unfairly negative and possibly simplistic light. How about the use of minimizers in hifiasm, a competitive OLC assembler?

**RESPONSE**: We rephrased the first paragraph to give a more unbiased description of OLC assembly. Moreover, we also mention the use of minimizers in Hifiasm.

OLC remains a popular approach for genome assembly, particularly when dealing with long-read technologies such as ONT and PacBio with higher error rates. In these cases, heuristics are often employed to mitigate the all-vs-all alignment bottleneck. For example, Hifiasm leverages minimizers to find overlaps among reads and perform read correction, making it capable of generating highly contiguous and haplotype-resolved assemblies of large eukaryotic genomes using PacBio HiFi reads.

26. “graph can be traversed by an Eulerian walk” this is in practice not performed. Contigs are just the simple paths of the simplified dBG. There is no Eulerian walk traversal.

**RESPONSE**: We revised the text as follows:

Indeed, following dBG construction, contigs (i.e., contiguous segments of the genome being assembled) are extracted from the simplified paths of the compacted and cleaned dBG.

27. “The number of unique k-mers and the possible overlaps between them is independent of sequencing depth” This is only true after a certain sequencing depth threshold, and even so, it is an exaggeration to state that it’s fully independent; but it can be stated that the number of nodes of error-cleaned dBGs plateau at higher sequencing depths (but in uncleaned dBGs, sequencing errors accumulate and generate more erroneous nodes). Also the overlaps are fully determined by the set of k-mers it, so there is never variability on the set of possible overlaps.

**RESPONSE**: We revised the text as suggested:

The number of nodes of an error-cleaned dBG saturates at higher sequencing depths but mainly depends on the size of the sequenced genome.

Thus, while the construction of dBGs is theoretically dependent on the genome's size, the inevitable inclusion of sequencing errors can inflate the graph size.

28. “to assemble a human genome in >4Mb contigs” this must be a typo, not Mb but million.

**RESPONSE**: Sorry for the typo. We have corrected it as: … assemble a human genome in >4 million contigs.

29. Figure 4 typos: “MGB”, “rust-mdGB”. But otherwise, this is a good figure.

**RESPONSE**: Thanks. We corrected Figure 4 (now Figure 5).

30. “Subsequently, a dBG is constructed in the minimizer space” [..] “ Following the minimizer-space dBG construction”. No: if the alphabet is nucleotides, this is not a minimizer-space dBG but a regular nucleotide-space dBG. You can instead write that “a dBG is constructed with minimizers as nodes”.

**RESPONSE**: We appreciate this point. We have removed the phrases “minimizer space” and “minimizer-space”.

31. In BCALM2 description: “Here, the parameter l is chosen to be less than k, ensuring that each k-mer possesses a minimizer at both its prefix and suffix”. This is not technically correct. There is nothing special about l, which follows the standard minimizer definition. The only novelty here is to make sure to select minimizers in k-1 prefixes of k-mers as well as select in k-1 suffixes of k-mers.

**RESPONSE**: We revised the sentence as suggested:

*BCALM2* [83] executes parallel graph compaction by categorizing k-mers into disk buckets utilizing s-mer minimizers selected in the k-1 prefixes and suffixes of the k-mers.

32. “Additionally, k-mers with neighboring hashing of minimizers” is vague

**RESPONSE**: We rephrase this paragraph to make it clear:

The algorithm integrates minimizers within the framework of Blocked Bloom Filters (BBFs), a data structure for membership query of elements in a set [86]. During the insertion of a k-mer into BBFs, Bifrost leverages the hash value of its minimizer to determine the appropriate BBF block for the k-mer. This ensures that sequences preceding or succeeding a particular k-mer are included in the same BBF block.

and “Since k-mers in different same BBF blocks” has an extra word.

**RESPONSE**: We apologize for the mistake. We removed the word “same”.

33. “However, none of these tools could scale up to many human genomes.” They can, with challenges, as per https://genomebiology.biomedcentral.com/articles/10.1186/s13059-023-03098-2

**RESPONSE**: We have reworded the statement to a more specific one:

Scaling up pangenomes to hundreds of human genomes remains computationally challenging and current efforts focus on developing methods able to accurately capture genomic variants across more genomes.

34. “It cannot call structural variations smaller than 50 bases” Neither can it call any variation (eg SNPs) smaller than 50 bases, not just structural.

**RESPONSE**: We removed the word “structural” to make the sentence accurate as suggested.

35. “Kraken2 determines which l-mers are minimizers by using a sliding window minimum algorithm.” Isn’t this the standard linear-time algorithm for computing minimizers? This is not exclusive to Kraken2, most minimizer-based tools use it.

**RESPONSE**: We revised the text as follows:

*Kraken2* uses the standard linear-time algorithm for computing minimizers in which s-mers are minimizers.

36. “we survey two algorithms that use minimizers for assembling the metagenomic data, namely Global clustering algorithm and MetaProb2.” The first program is not an assembly program and should probably not be included in this section.

**RESPONSE**: That’s a good point. The sentence was not accurate, although “Global clustering algorithm” is useful as a preprocessing technique in metagenomics assembly. As suggested, we agree to remove this tool from the manuscript since especially the manuscript is becoming quite long.

In this section, we survey *MetaProb2*, an algorithm that uses minimizers for assembling the metagenomic data.

37. "First, reads are grouped based on their overlap that they share a common subsequence using minimizers (assumed to be of the same species) and then assembled using miniasm (de novo assembly tool) and minimap2 (the mapping tool; Section 3.1)," This sentence needs to be revised for technical accuracy, at two locations: the overlap part, and *minimap2* is not an assembler.

**RESPONSE**: Indeed minimap2 is not an assembler. *Minimap2* is used as part of the assembler to map reads against each other and to find overlapping reads. We revised the text as follows:

First, reads are grouped based on their common subsequence using *minimap2* (assumed to be of the same species) and then assembled using long-read de novo assembly algorithms, such as *miniasm*.

We would be happy to clarify it further if the description of the overlapping part is not clear.

38. “with highly divergent sequences with substitutions and indels where k-mer based approaches are susceptible” Unsure here is susceptible is the right adjective. Did you mean, prone to failure?

**RESPONSE**: We revised the sentence as suggested:

..where *k-mer*-based approaches are prone to fail.

39. In the conclusion, “This is achieved by representing sequences with shorter subsequences” is vague, as sequences are not transformed into an equivalent representation, but rather sketched into a potentially highly lossy representation.

**RESPONSE**: We acknowledge that the representation is lossy. The claim that shorter subsequences, minimizers, represent the original sequence, is correct in one way and has practical benefits. To address this, we revised the text as follows:

This is achieved by creating “sketches” of sequences that occupy less space compared to the sequences themselves.

40. In the conclusion, “from combining insights of algorithmic functions” has unclear meaning.

**RESPONSE:** We removed this phrase.

41. In the conclusion, “Minimizers are used to process data in a way that is efficient and suitable for numerical operations. These techniques have certain trade-offs and limitations such as probabilistic approximate results and hash functions with the potential for collisions.” is vague and appears to confuse multiple independent concepts, as minimizers do not have to be combined with hashing.

**RESPONSE:** Since the phrasing did not convey important information (and the manuscript now becomes lengthy), we decided to remove this.

# Reviewer #2:

This is a well-written review focused on minimizers, with a reasonably substantial discussion of k-mers. I learned some new things from it, which was nice! (I am aware I am coming in as a second round reviewer!)

**RESPONSE**: We thank the reviewer for this highly positive assessment. We are glad the reviewer found the manuscript informative, as this was our purpose.

I think this is already quite good, but could be improved by adding some specific content above section 3. In brief, I found the transition between section 2 (here's what minimizers are) and section 3 (here's how they're used) to be pretty abrupt. From a teaching/learning perspective, the transition could be improved with a simple example and a diagram - the minimap example seems just about perfect for this!

**RESPONSE:** We have added the following figure to the manuscript as suggested.

I would also welcome a little bit more explanation of how minimizers differ from using all the k-mers. I can intuit a lot of it from the discussion, but a simple summary statement would be useful. It's entirely possible it's there and I missed it.

**RESPONSE:** Thank you for this suggestion. We have included a short summary at the end of the *k-*mers section to introduce minimizers:

One such way that has emerged is minimizers, which is based on *k*-merization of the data. Minimizers achieve faster processing and reduced memory usage by working with only a subsample of the *k*-mers.

# Reviewer #3:

The manuscript is a survey about minimizers' applications in genomics. Minimizers are sketching algorithms that retain a (typically, small) sample of the kmers from a sequence S, hence permitting to save considerable memory and computing time. They found several important applications, as described in the manuscript. In my opinion the survey offers a quick start into the concept of minimizers and overview some important applications. It is not, however, particularly technical but I would not regard this as a problem given that it is useful to also give general overviews without too much technicalities that can prevent from getting the core intuitions. Thus, I'm in favour of accepting this manuscript *provided that* the authors include some changes that I list below.

**RESPONSE**: We appreciate the reviewer’s positive assessment of the manuscript.

Major: First, there are some corrections to be made (see "Minor", below).

**RESPONSE**: We have addressed each suggestion.

Second, I suggest another important application of minimizers, that is: representing de Bruijn graphs (dBGs). This should be definitely added to Table 1. Currently, the manuscript talks about dBGs when discussing de novo assembly in Section 3.3 and it seems to mix the two concepts of "assembly" and "dBG construction". These two things are related, sure, but they are not the same. A dBG can help the process of de novo assembly but it is not the only approach. So, I would suggest to distinguish between the two and to have a separate Section -- before 3.3 -- headed "Representing de Bruijn graphs". As you mention, dBGs are fundamental for many applications in genomics.

**RESPONSE**: That is a very good suggestion. We added a new section titled “3.3 Representing de Bruijn graphs” and updated the section “3.4 De novo genome assembly” accordingly.

This section should include recent development made in using minimizers to compactly represent dBGs and colored dBGs. Currently, you only mention Bifrost which is not the state of the art anymore. Another approach based on minimizers is Blight [6], although the current state of the art is an approach called SSHash [1,2]. SSHash represents a (possibly, weighted) de Bruijn graph using random minimizers and minimal perfect hashing. It has also been used to build positional indexes over collection of references [3], and to represent colored compacted de Bruijn graphs [4,5]. For example, Fulgor [4,5] is a colored de Bruijn graph index based on minimizers that solves the problem of read (pseudo) alignment, hence could also be mentioned in Section 3.1.

**RESPONSE**: Thank you for pointing out this important insight. We have included these methods in the new section “3.3 Representing de Bruijn graphs”.

Minor:

- Abstract: "minimizers, allows" --> "minimizers allow"

**RESPONSE**: Here we considered “minimizers” as extra information, on top of the subject of the sentence “One such advance”. We now updated the abstract based on Editorial suggestions.

The exponential increase in sequencing data calls for conceptual and computational advances to extract useful biological insights. One such advance, minimizers, allows for reducing the quantity of data handled while maintaining some of its key properties. … We also touch on alternative data sketching techniques including universal hitting sets, syncmers, or strobemers. Minimizers and their alternatives have rapidly become indispensable tools for handling vast amounts of data.

- Figure 1: what is the order being used here? Lexicographic? Then the orange kmer selected should not be "GAC" but "ACC".

**RESPONSE**: Thanks. We now consider the lexicographic order and updated the figure with “ACC” as the orange minimizer instead of GAC.

- Section 2.3, when you talk about orders, I would mention (if not done already) that in practice we use a pseudo-random hash function to implement the order (hence, it is essentially random) because it performs better than lexicographic order and allows to compare the hashes of the kmers rather than the actual strings themselves.

**RESPONSE**: That’s a very good point. To address this, we have added the following to Section 2.4.

Random ordering can result in a lower density than that of the lexicographic ordering. Thus, random ordering is usually used in practice and is implemented with pseudo-random hash functions.

- caption of Figure 2: "(≥w+k-1),it..." --> "(≥w+k-1), it..." (missing blank space).

**RESPONSE**: We have added the space.

- Section 2.4: "A hashing function" --> "a hash function"

**RESPONSE**: We have altered the phrase.

"which reduces the memory ... output numbers smaller than k." First, I do not get what you mean; please reformulate. Second, perhaps you meant "smaller than |\Sigma|^k".

**RESPONSE**: Thank you for the comment, which was also raised by Reviewer #1. We have corrected the paragraph as below:

A hash function transforms a given string of arbitrary size (*k*-mers in our case) into a fixed-sized value. In doing so, the resulting representation will typically occupy less space [29] (e.g., hashing into 32-bit values [30] results in space saving for genomic *k*-mers for *k*>16 assuming a 2-bit encoding per nucleotide).

- At the end of Section 2.4, there are problems with the base of the logarithms. It is not \Sigma, but |\Sigma|, i.e. the size of the alphabet (e.g., 4 for DNA).

**RESPONSE**: Thanks. We have corrected both occurrences: log_|Σ|_(w+1) … log_|Σ|_(w)

Also, the Big-Oh term is not O(1/w) but o(1/w), that is a *little-oh* not a Big-Oh. This makes a large difference, both in theory and practice.

**RESPONSE**: We have corrected the sentence based on the reference.

Zheng et al. provided explicit conditions only on *k* (*i.e.*, *k*≥(3+ε)log|Σ|(w+1)) for the expected density of 2/(w+1)+o(1/w).

- When you talk about BCALM2, you should definitely also mention GGCAT [7] which is a substantial improvement over BCALM2 (much faster in practice).

**RESPONSE**: Thanks for pointing out this paper to us. We added a paragraph on GGCAT in the new section “3.3 Representing de Bruijn graphs”.

- Please, use a pageref when referring to Table 1.

**RESPONSE**: pageref is usually used in Latex and we used Google Docs to prepare this manuscript. Now we use Bookmark to connect where the table appears (now at the end of the manuscript as requested by the Editor during the peer-review process). If the manuscript gets accepted, we will make sure of this during the proofreading.

- Section 4.2: "Finding polar set is shown to be NP-hard but a heuristic algorithm is proposed to identify them in linear time." -- This sentence is contradictory: the problem is NP-hard, meaning no polynomial-time algorithm exist (assuming P is not equal to NP), hence you can only find an *approximation* to polar sets.

**RESPONSE**: Thanks for highlighting this point. We have revised it as below:

… but a heuristic algorithm is proposed to identify their approximations in linear time.

- Section 4.3: at the end of the section you should mention that while Miniception does not require the storage of a precomputed set, sequence-specific schemes can essentially reach the lower bound of 1/w in density whereas Miniception cannot.

**RESPONSE**: we have added the following sentence to the paragraph to convey the point.

While having these advantages in time and memory performance, the lower bound of the resulting sketch (1.67/(w+1)) is higher than the theoretical lower bound (1/w), which can be achieved using UHS or Polar Sets.

- Section 5: "However, as algorithms and computational hardware...less significant." First, I do not understand why reference [33] is cited. It makes no sense to me. Second, I would not dare to make such a statement. Computational improvements are far more likely to be coming from algorithmic design and not from hardware improvement.

**RESPONSE**: Thanks for bringing up this point. We had mentioned reference 33 (doi.org/10.1093/bioinformatics/btae045) as an example of recent algorithmic advancement since it was published very recently. Regarding the second comment, we think hardware improvement over the decades made it possible to have machines with larger memory and computation power capacities. One could also mention the example of GPU which was for rendering images but being repurposed for computation. We meant here that if we were less limited in memory and process resources, it would be easier to run algorithms on a large scale, and the issue would become less significant. To avoid possible different interpretations and save space, we have removed this sentence.

- Section 5: the last sentence ("Other lines of research...") logically belongs to the previous paragraph where you talk about efforts spent in reducing the density of minimizers.

**RESPONSE**: We have revised the text as suggested. “Specifically, lines of research ...”

References

[R1] Sparse and Skew Hashing of k-mers, Giulio Ermanno Pibiri, Bioinformatics, 2022

[R2] On Weighted k-mer Dictionaries, Giulio Ermanno Pibiri, Algorithms for Molecular Biology, 2023

[R3] Spectrum preserving tilings enable sparse and modular reference indexing, Fan et al., In 27-th International Conference on Research in Computational Molecular Biology, 2023

[R4] Fulgor: A fast and compact k-mer index for large-scale matching and color queries

Fan et al., Algorithms for Molecular Biology, 2024

[R5] Meta-colored compacted de Bruijn graphs, Pibiri et al., In 28-th International Conference on Research in Computational Molecular Biology, 2024

[R6] BLight: Efficient exact associative structure for k-mers, Marchet et al., Bioinformatics, 2021

[R7] Extremely fast construction and querying of compacted and colored de Bruijn graphs with GGCAT, Cracco, Andrea, and Alexandru I. Tomescu, Genome Research, 2023

**RESPONSE**: Thanks for providing the references, which we found useful. We have included these references in the manuscript.

**3^rd^ round**

**Reviewer 1**

The response has been appropriate for my comments. I leave it to Reviewer 3 to check accuracy of the requested new sections (in my opinion, the paper was long enough already without them).

**Reviewer 3**

In this revision the authors made all the changes I suggested. I think the manuscript is good as is and it will serve as a primer on minimizers' applications. I have some minor comments:

- in Sec. 2.4: "While the optimal minimum is 1/w, it is not achieved in practice [22]" -- you should mention that recently, here https://drops.dagstuhl.de/entities/document/10.4230/LIPIcs.WABI.2024.11, it was shown that the mod-minimizer tends to the optimal density 1/w when k is large.
I see you already cite this paper, but only in its biorxiv version. Please, check all references that are pre-prints to see if they have been published.

- in Sec. 3.3 when you talk about Fulgor and say "resulting in an index with size of only 70GB" referring to the 150K Salmonella pangenome, you can also note that this was further reduced to < 7.5GB (almost 10X improvement) in paper [80].
